# Genomic Characterization of *Listeria monocytogenes* Strains Involved in a Multistate Listeriosis Outbreak Associated with Cantaloupe in US

**DOI:** 10.1371/journal.pone.0042448

**Published:** 2012-07-31

**Authors:** Pongpan Laksanalamai, Lavin A. Joseph, Benjamin J. Silk, Laurel S. Burall, Cheryl L. Tarr, Peter Gerner-Smidt, Atin R. Datta

**Affiliations:** 1 Center for Food Safety and Applied Nutrition, FDA, Laurel, Maryland, United States of America; 2 Division of Foodborne, Waterborne, and Environmental Diseases, Centers for Disease Control and Prevention, Atlanta, Georgia, United States of America; University of Illinois at Chicago College of Medicine, United States of America

## Abstract

A multistate listeriosis outbreak associated with cantaloupe consumption was reported in the United States in September, 2011. The outbreak investigation recorded a total of 146 invasive illnesses, 30 deaths and one miscarriage. Subtyping of the outbreak associated clinical, food and environmental isolates revealed two serotypes (1/2a and 1/2b) and four pulsed-field gel electrophoresis two-enzyme pattern combinations I, II, III, and IV, including one rarely seen before this outbreak. A DNA-microarray, *Listeria* GeneChip®, developed by FDA from 24 *Listeria monocytogenes* genome sequences, was used to further characterize a representative sample of the outbreak isolates. The microarray data (in the form of present or absent calls of specific DNA sequences) separated the isolates into two distinct groups as per their serotypes. The gene content of the outbreak-associated isolates was distinct from that of the previously-reported outbreak strains belonging to the same serotypes. Although the 1/2b outbreak associated isolates are closely related to each other, the 1/2a isolates could be further divided into two distinct genomic groups, one represented by pattern combination I strains and the other represented by highly similar pattern combinations III and IV strains. Gene content analysis of these groups revealed unique genomic sequences associated with these two 1/2a genovars. This work underscores the utility of multiple approaches, such as serotyping, PFGE and DNA microarray analysis to characterize the composition of complex polyclonal listeriosis outbreaks.

## Introduction

*Listeria monocytogenes*, a Gram-positive foodborne bacterial pathogen, is the causative agent for human and animal listeriosis. The invasive form of human listeriosis (Inv) is a disease predominantly affecting immuno-compromised people, the elderly, neonates and pregnant women and is characterized by septicemia and/or meningitis in non-pregnancy-associated cases, fetal loss, premature labor and neonatal septicemia or meningitis in pregnancy-associated cases with high case-fatality ratios (∼20–30%) [1]–[4]. The Centers for Disease Control and Prevention (CDC) estimates that approximately 1600 cases of Inv listeriosis and 255 deaths occur annually in the United States [5]. The febrile gastroenteritis (FG) form of listeriosis affects otherwise healthy people and is characterized by self-limiting fever, aches, fatigue, nausea and diarrhea [6], [7]. The cause behind such disparate outcomes is yet to be determined. The burden of FG listeriosis is unknown because *L. monocytogenes* is not routinely cultured from stool in clinical laboratories.

Although the majority of the large listeriosis outbreaks detected in the United States have been associated with the consumption of ready-to-eat frankfurters and deli meats, as well as milk and dairy products, outbreaks due to consumption of produce (e.g., cabbage, sprouts, pre-chopped celery) also have been previously reported [8]. The organism has been isolated from soil, water, vegetation, and a variety of food processing environments [9]–[11]. Raw vegetables and fruits, including cantaloupe, have been found to contain *L. monocytogenes* [12]–[14]. Also, a case-control study identified “eating melons at a commercial establishment” as a risk factor for sporadic listeriosis [15]. However, melon consumption had never been implicated in any listeriosis outbreak in U. S. until the investigation of a multistate outbreak associated with cantaloupe began on September 2, 2011 [16].

From August 12 to November 1, 2011, 146 outbreak-associated cases of invasive listeriosis were diagnosed among residents of 28 states in the USA. Thirty deaths and one miscarriage were reported during the outbreak, which was the largest number of fatalities due to an outbreak of foodborne listeriosis in the United States [17]. All cases of invasive listeriosis were culture-confirmed. Nearly all clinical isolates of *L. monocytogenes* were obtained from samples of blood or cerebrospinal fluid. The outbreak strains consisted of serotypes 1/2a and 1/2b and belonged to four distinct pulsed-field gel electrophoresis (PFGE) pattern combinations (PCs) I, II, III, and IV. Investigation by local, state and federal public health and regulatory agencies identified *L. monocytogenes* outbreak strains on cantaloupes collected from grocery stores, ill person's home, and whole cantaloupes collected from cold storage and the packing facility environment; environmental swabs collected at the facility also identified *L. monocytogenes* outbreak strains [17]. Investigation of the packing facility identified several factors, including inadequate cleaning and sanitization of the processing equipment, lack of a pre-cooling step before cold storage, and packing facility design, as most likely contributors to the introduction, spread, and growth of *L. monocytogenes* in the cantaloupe [18]. This was the first report of a listeriosis outbreak caused by the consumption of fresh fruit in the home environment.

Although *L. monocytogenes* can be classified into 13 serotypes [19], the vast majority of listeriosis outbreaks are caused by strains of three serotypes 1/2a, 1/2b and 4b. Of these serotypes, 4b accounts for most of the major outbreaks although a few recent outbreaks have been caused by strains of serotypes 1/2a and 1/2b [20]–[24]. The 2011 cantaloupe outbreak is one of the few reported outbreaks where multiple serotypes have been involved and multiple PFGE types have been implicated [16]. To gain further understanding of these outbreak strains, we have analyzed a total of 35 outbreak associated isolates obtained from cantaloupe, the cantaloupe processing environment and patients by serotyping and by two-enzyme PFGE typing. Since the DNA microarray technology has been shown to be a versatile method to quickly assess gene contents and genomic architecture in several organisms [25]–[28], we also analyzed 16 isolates, a representative sample of these outbreak isolates, using a custom DNA microarray developed in FDA. The microarray – *Listeria* GeneChip® [28], contains sequence from 24 *L. monocytogenes* genomes available in public databases as of 2009. Using *Listeria* GeneChip®, we have already shown that the outbreak strains of *L. monocytogenes* can be further classified into different genomic groups or genovars and it could also identify epidemic clones and could further distinguish the individual outbreak strains and food and clinical isolates [28]. In this communication, we report the analysis of a representative sample of the outbreak associated isolates and determine the genetic relatedness of these isolates with one other and with strains from previously reported outbreaks. Using microarray data, we also identified individual genetic footprints of the outbreak strains and show how they differ from each other and from previous outbreak strains involving same serotypes. Finally, our data clearly established the usefulness of multiple molecular subtyping tools to characterize the full scope of a polyclonal, complex outbreak.

## Materials and Methods

### Serotyping of *L. monocytogenes* isolates

*L. monocytogenes* isolates were identified using methods described in the Bacteriological Analytical Manual [29]. Serotypes were determined by a combination of multiplex PCR and antisera agglutination as described previously [30]. Briefly, overnight brain heart infusion agar (BHI-A) cultures were used to make lysates used for multiplex PCR analysis as well as antisera agglutination assays using Difco Listeria O antisera types 1 and 4 (BD Diagnostic Systems, Sparks, MD). The agglutination assay protocol was modified by suspending the bacteria in approximately 1 mL of FA buffer (BD Diagnostic Systems) and then followed as described in the manufacturer's protocol. A few of the isolates were also serotyped by using DK anti-sera (Denka Seiken Co., Tokyo, Japan) according to the manufacturer's instructions.

### PFGE analysis of *L. monocytogenes* isolates

*L. monocytogenes* isolates were typed following the standard PulseNet procedure using restriction enzymes *AscI* and *ApaI* (www.pulsenetinternational.org) [31]. PFGE profiles were named and compared against those in the CDC PulseNet database as previously described [32].

### *L. monocytogenes* strains and preparation of genomic DNA for hybridization

Cantaloupe outbreak isolates (Table 1) were grown in brain heart infusion (BHI) broth and/or BHI agar at 37°C. Isolates used in the comparison of genomic contents were obtained from various sources as described by Laksanalamai et al, 2012 [28]. Genomic DNA was isolated from 10 ml of cultures grown overnight in a shaking incubator at 170 rpm using the Qiagen DNeasy Blood and Tissue kit (Qiagen, Valencia, CA) with modifications described previously [28]. 10 µg of the genomic DNA were fragmented by 0.2 unit DNaseI (Promega, Madison, WI) at 37°C for 10 minutes in a 40 µl reaction volume containing 1× One-Phore-All buffer (GE Healthcare, Waukesha, WI), followed by heat-inactivation at 95°C for 10 minutes. The fragmented DNA was 3′-end labeled with 2 nM of biotin-11-ddATP (ABI) and 60 units of terminal transferase (Promega, Madison, WI) at 37°C for 4 hours. The labeled product was then used for hybridization onto the Listeria GeneChip®.

**Table 1:** *Listeria monocytogenes* strains used in this study.

Isolate	Alternate designation	Source	PFGE pattern combination^a^	Serotype
LS667	713431 10-B	Food	II	1/2b
LS668	713432 30-C	Environmental	IV	1/2a
LS669	713431 10B-E	Food	II	1/2b
LS670	713431 6B-B	Food	II	1/2b
LS671	713431 7-B	Food	II	1/2b
LS672	713431 8-B	Food	II	1/2b
LS673	713432 20-C	Environmental	II	1/2b
LS674	713432 21-C	Environmental	II	1/2b
LS675	713432 22-C	Environmental	II	1/2b
LS676	713432 23-C	Environmental	II	1/2b
LS677	713432 24-A	Environmental	II	1/2b
LS678	713432 26-C	Environmental	II	1/2b
LS679	713432 27-C	Environmental	II	1/2b
LS680	713432 28-C	Environmental	II	1/2b
LS681	713432 29-A	Environmental	II	1/2b
LS682	713432 33-C	Environmental	II	1/2b
LS683	643701-1	Food	II	1/2b
LS684	643701-10	Food	II	1/2b
LS685	643701-2	Food	II	1/2b
LS686	643701-3	Food	II	1/2b
LS687	643701-5	Food	II	1/2b
LS688	643701-6	Food	II	1/2b
LS689	643701-7	Food	II	1/2b
LS690	643701-8	Food	II	1/2b
LS691	643701-9	Food	II	1/2b
LS692	713432 13-C	Environmental	III	1/2a
LS693^b^	133201-1	Shrimp	IV	1/2a
LS740	HUM-2011024433	Patient	II	1/2b
LS741	HUM-2011024476	Patient	II	1/2b
LS742	HUM-2011024899	Patient	I	1/2a
LS743	HUM-2011024909	Patient	I	1/2a
LS745	HUM-2011025927	Patient	III	1/2a
LS746	HUM-2011026221	Patient	III	1/2a
LS744	HUM-2011026129	Patient	IV	1/2a
LS747	HUM-2011026798	Patient	IV	1/2a

a PFGE pattern combinations were assigned based on the *AscI* pattern and the *ApaI* pattern as described in Figure 1 and Table 2.

b Non-outbreak related.

### Array hybridization, washing, staining and scanning

Hybridizations of the genomic DNA isolated from cantaloupe outbreak strains were performed according to the Affymetrix GeneChip® Expression Analysis Technical Manual [33]. Hybridization reactions containing 10 µg of labeled fragmented DNA, 100 mM MES, 1M(Na^+^), 20 mM EDTA, 0.01% Tween-20, 50 pM control oligoB2 (Affymetrix, Santa Clara, CA), 0.1 mg/ml herring sperm DNA (Promega), 7.8% dimethylsulfoxide (DMSO) (Sigma, St. Louis, MO), were heated at 95°C for 1 minute followed by incubation at 45°C for 5 minutes, prior to hybridizing onto the Affymetrix *Listeria* GeneChip® at 45°C with rotation (60 rpm) for 16 hours in a hybridization oven. The wash and staining procedures were carried out on an Affymetrix FS-450 fluidics station using the mini_prok2v1_450 fluidics script as described by GeneChip® Expression Analysis Technical Manual with the slight modification previously described [28]. Arrays were scanned using a GeneChip® Scanner 3000 7G with GCOS v1.4 software.

### Parsing CEL files, probe-set summarization methods, data analysis tools, and genomic relationship analysis

All Affymetrix CEL files generated in this study were parsed and analyzed using algorithms including MAS5.0 [27], [34], [35] with a Tau value as reported previously [28]. Robust Multi Array (RMA) method to identify summarized probe-set intensities was implemented by the Affy package of R and Bioconductor [36]–[38].

The gene present/absent binary nucleotide calls based on the gene contents as T (present) and A (absent), were concatenated for each strain, such that a 18,630 bp sequence was generated to represent the gene content [28]. Non-phylogenetically informative [39] probe-sets which were either present or absent in all of the tested strains were eliminated from the analysis. The parsimonious informative sites were identified from the concatenated gene content sequences of each strain using Splitstree 4.11.3 [40]. A neighbor-net or neighbor joining phylogeny highlighting the distribution of *L. monocytogenes* serotype 1/2a and 1/2b was constructed using the uncorrected *p*-distance in Splitstree 4.11.3.

## Results and Discussion

### Serotyping of *L. monocytogenes* isolates

All isolates showed a positive agglutination reaction with Difco Type 1 antisera. Combining these results with the multiplex PCR results (data not shown) allowed us to make a final serotype determination within 24 hours of inoculating the initial BHI-A overnight culture. The multiplex, PCR-based serotypic determinations of several strains were independently confirmed by a standard antisera-based serotyping protocol using the Denka-Seiken commercial kit (data not shown). Thus, the multiplex PCR-agglutination protocol allowed us to rapidly and accurately determine the serotypes for the outbreak isolates. These analyses confirmed that this outbreak was a multi-strain and multi-serotype outbreak, involving two distantly related serotypes [28] and, to best of our knowledge, this may be the first reported listeriosis outbreak involving a single commodity contaminated by two different serotypes of *L. monocytogenes*. Although the majority of recorded invasive listeriosis outbreaks were caused by serotype 4b, serotype 1/2a and 1/2b have been reported more frequently in recent years. Serotype 1/2a and 1/2b isolates have also been involved in several FG listeriosis outbreaks [41].

### Comparison of *L. monocytogenes* outbreak PFGE pattern combinations in the CDC PulseNet national database

Molecular subtyping is one of the crucial tools for outbreak investigation. Since 2001 standardized PFGE has become the gold standard for establishing genetic relatedness among bacterial isolates obtained from foods, clinical samples and the environment [42], [43]. With the advent of the national PulseNet network, the PFGE profiles of clinical and suspected food isolates are routinely uploaded into the national database and analyzed for PFGE pattern similarity using Bionumerics software [43]. This information has been successfully utilized in many outbreak investigations leading to a reduction in foodborne listeriosis [44].

PFGE patterns (*AscI* and *ApaI*) from selected outbreak strains (Table 1) were compared in the CDC PulseNet database. There were >12,700 *L. monocytogenes* isolates with *AscI* and *ApaI* patterns in the national database before this investigation. The PFGE patterns of the outbreak strains matched one of four different PCs in the national database and are termed I, II, III, and IV (Table 2 and Fig. 1). By definition, all patients were infected with one of the four outbreak-associated PCs, and infections with PC I (n = 48) and PC II (n = 40) were more frequently represented than PC III (n = 28) and PC IV (n = 30). Although not empirically demonstrated, the frequency distribution of PFGE patterns among clinical isolates (and isolates from other sources) may have resulted randomly from arbitrary colony picks on culture plates where >1 outbreak strain had grown (i.e., co-infections with multiple strains and serotypes is possible). Two of the PCs (III and IV) shared the same *ApaI* pattern name (GX6A12.0001); however, the *AscI* patterns (GX6A16.0099 and GX6A16.0001, respectively) differed by a band shift of ∼40 kb possibly due to the presence of a phage in PC IV isolates (Knabel et al., unpublished data). The PCs assigned to each isolate examined are shown in Table 1. Evaluation of the results showed that all isolates belonging to PC II were serotype 1/2b and isolates with PC I, III and IV were serotype 1/2a. This is one of the few reported listeriosis outbreaks where more than one PFGE profiles has been found to be associated with the outbreak.

**Figure 1:**
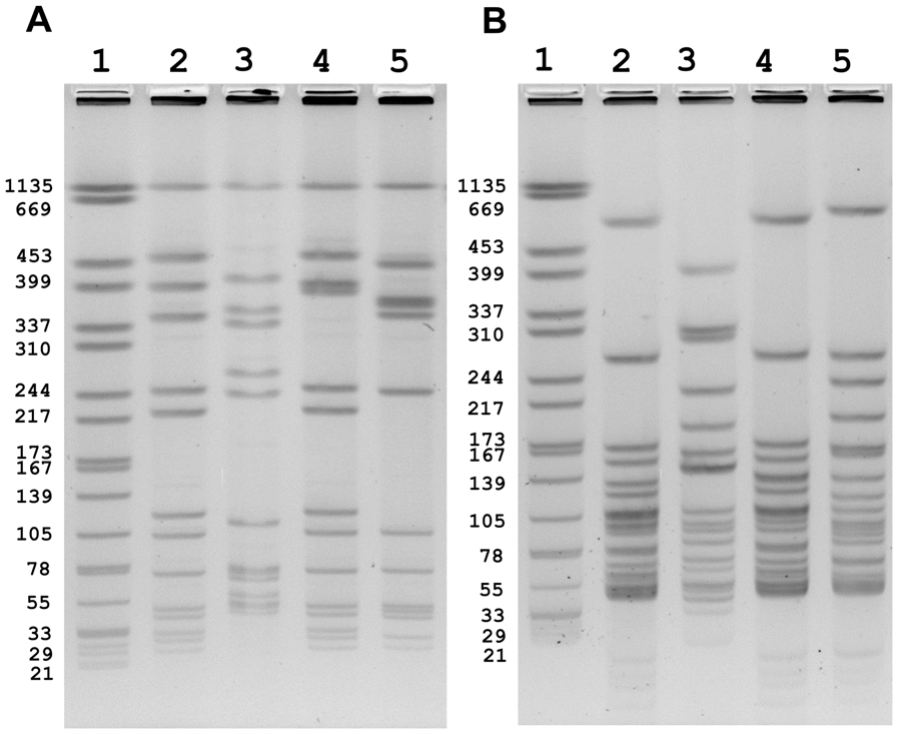
PFGE profiles *of Listeria monocytogenes* cantaloupe outbreak strains. *AscI* patterns are shown in panel A: lane 2: GX6A16.0099 (III); lane 3: GX6A16.0019 (II); lane 4: GX6A16.0001 (IV); lane 5: GX6A16.0029 (I) and *ApaI* patterns in panel B lane 2: GX6A12.0001 (III); lane 3: GX6A12.0227 (II); lane 4: GX6A12.0001 (IV); lane 5: GX6A12.0069 (I). Lane 1 in both panels is *Salmonella braenderup* H9812 standard (digested by *XbaI*). The Roman numerals represent the PFGE pattern combination.

**Table 2:** PFGE pattern combinations associated with the outbreak.

PFGE Pattern combination	*Asc*I/*Apa*I PulseNet pattern names
I	GX6A16.0029/GX6A12.0069
II	GX6A16.0019/GX6A12.0227
III	GX6A16.0099/GX6A12.0001
IV	GX6A16.0001/GX6A12.0001

**Figure 2:**
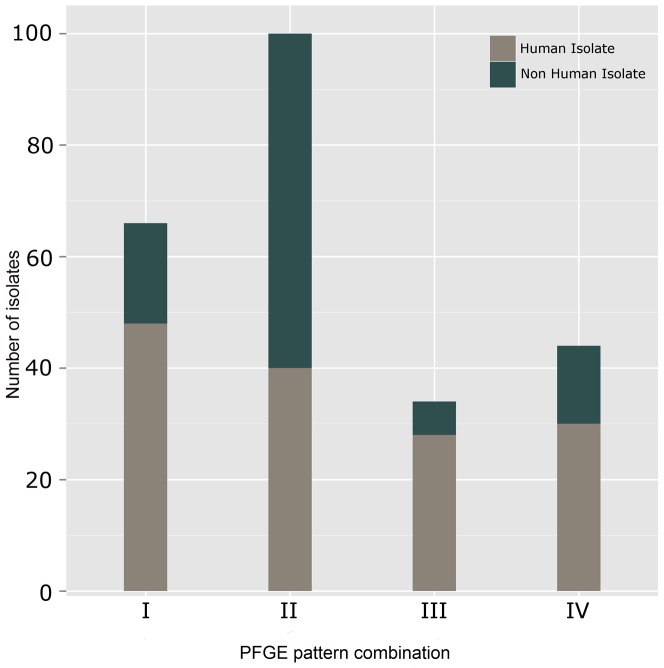
Distribution of *Listeria monocytogenes* isolates producing PFGE pattern combinations. I- IV from human (grey) or nonhuman (green) sources. Only isolates from August 25–November 15, 2011 (duration of the outbreak) are included.

The number of human isolates in each PC ranged from 30 to 48; whereas, the number of non-human isolates (cantaloupe and environmental swabs of the cantaloupe processing facility) of these PCs in the database ranged from 6 to 60 with most of these isolates belonging to PC II (Fig. 2). Interestingly, PC II is relatively new in the database with the first nonhuman isolate uploaded in August 2010 and the first human isolate uploaded as a part of this outbreak. This could indicate emergence of a new strain of *L. monocytogenes* with potential for increased survival capability in foods and/or environment.

### Microarray analysis of the *L. monocytogenes* cantaloupe outbreak related isolates

It is clear from the above paragraphs that the cantaloupe outbreak was caused by at least three strains belonging to two different serotypes. Four PCs were identified, of which two were closely related. In order to further characterize these strains at the genomic level, we chose eight isolates from food or the food processing environment and eight isolates from patients for microarray analysis (Table 1).

**Figure 3:**
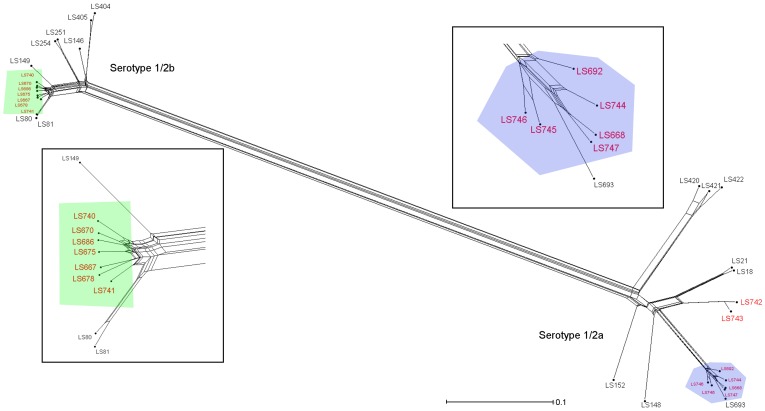
Relatedness analysis of the compatible parsimony informative genes from the 15 cantaloupe associated outbreak isolates, a shrimp isolate (LS693) and 15 *L. monocytogenes* isolates from several unrelated listeriosis episodes/outbreaks. The tree was generated from the concatenated gene contents using neighbor joining with the uncorrected p distance. The numbers in red color indicate the cantaloupe associated outbreak and the shrimp isolates. The boxes indicate the close-up portions of the cantaloupe outbreak strains (green; serotype 1/2b and blue; serotype 1/2a). Scale bar represents number of gene differences (present or absent) per gene site. The Roman numerals represent the PFGE pattern combination.

The gene contents (present/absent) of these *L. monocytogenes* strains were identified from the microarray results using the Affy package in R-Bioconductor. The gene contents of the cantaloupe outbreak strains from both serotypes 1/2a and 1/2b were compared with the unrelated strains, which are ones we have previously characterized [28] and an unrelated isolate from shrimp (LS693) with the same PFGE pattern as PC IV. Among 18,360 probe-sets on the *Listeria* GeneChip®, 7,823 probe-sets were conserved, which left 4,041 (21.7%) and 6,766 probe-sets (36.3%) as present and absent, respectively. A neighbor-net tree, constructed based on the gene content analysis, clearly separated these cantaloupe outbreak and unrelated strains according to their serotypes (Fig. 3). Previous work showed that the 1/2a strains were well separated from 1/2b strains with the latter forming a much more closely related group [28]. Within each group, the cantaloupe-associated strains (highlighted in red, Fig. 3) were found to form their own groups indicating that these strains are genetically distinct from the previously reported serotype 1/2a and 1/2b outbreak and sporadic strains.

### Cantaloupe outbreak associated isolates of serotype 1/2a

**Figure 4:**
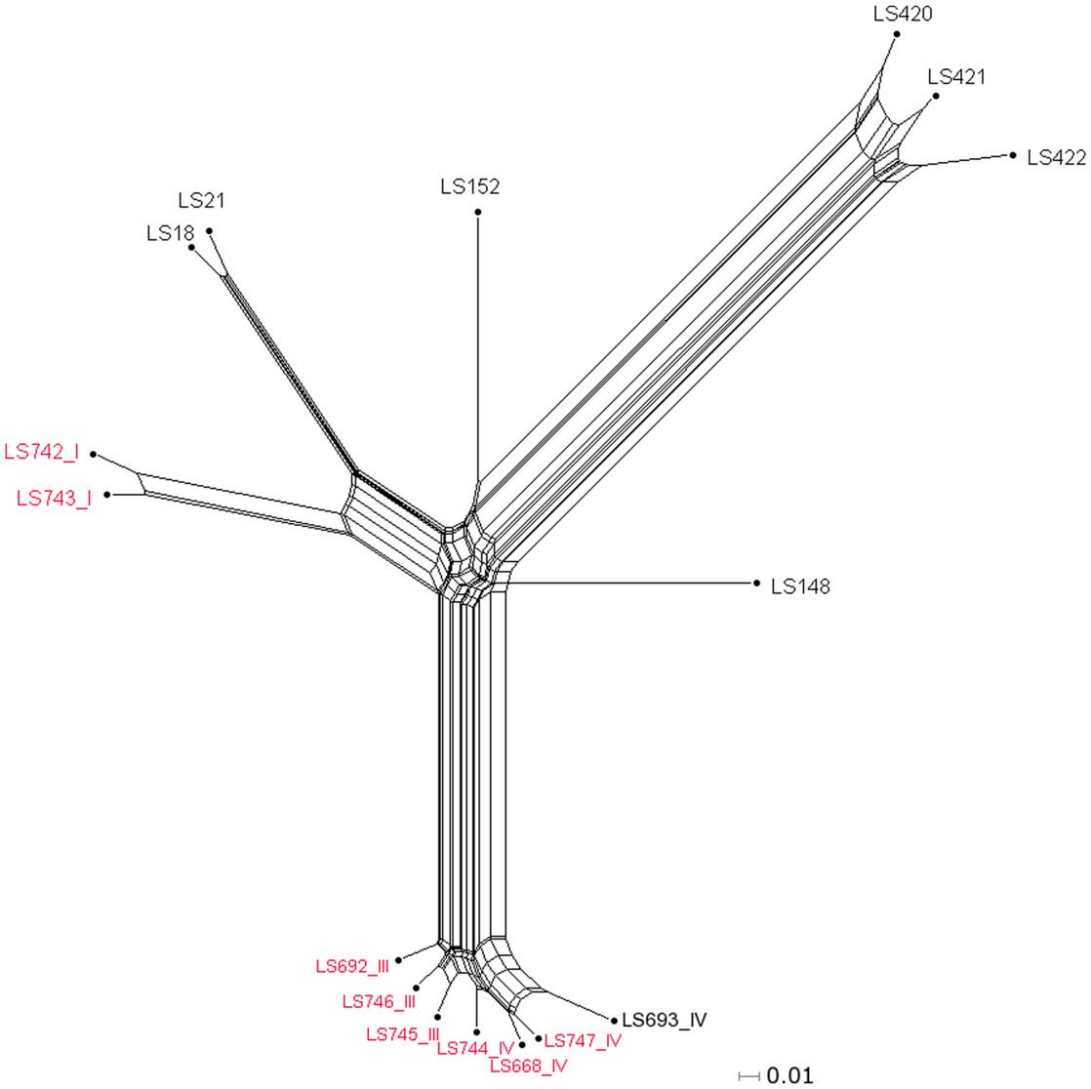
Relatedness analysis of the compatible parsimony informative genes from the 9 cantaloupe associated outbreak isolates, a shrimp isolate (LS693) and 7 unrelated *L. monocytogenes* isolates belonging to serotype 1/2a [**28**]. The tree was generated from the concatenated gene contents using neighbor joining with the uncorrected p distance. The numbers highlighted in red indicate the cantaloupe associated outbreak and the shrimp isolates. Scale bar represents number of gene differences (present or absent) per gene site. The Roman numerals represent the PFGE pattern combination.

**Figure 5:**
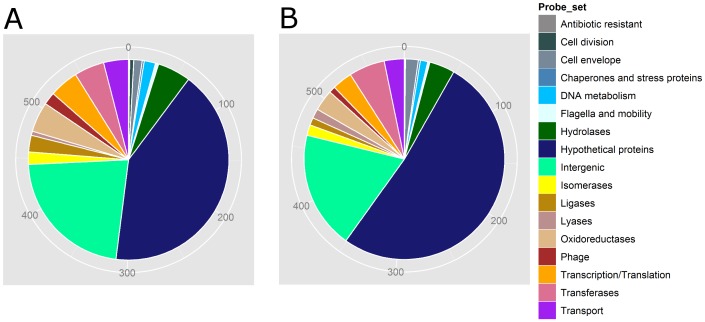
Diagrammatic representation of the distribution of unique probe-sets of the serotype 1/2a 2011 cantaloupe outbreak strains from different PFGE pattern combination groups based on their functions. A) Probe sets uniquely present in the strains from the PFGE pattern combination I and absent in both PFGE pattern combination III and IV. B) Probe sets uniquely present in the strains from the PFGE pattern combination III and IV and absent in the PFGE pattern combination I. The numbers of probe-sets were indicated in the periphery of the pie-chart.

We examined the genetic contents of serotype 1/2a cantaloupe outbreak strains representing all three different PCs (I, III and IV) using MAS5.0 algorithm. Gene content comparison among these cantaloupe outbreak isolates, an unmatched shrimp isolate (LS693), and previously reported strains [28] from the serotype 1/2a revealed that the cantaloupe outbreak isolates are clearly divided into two distinct genomic groups or genovars (Fig. 4). Among the 1/2a strains we characterized, there were 13,916 conserved probe-sets, which encompassed 74.7% of total probe-sets. Strains LS742 and LS743, two clinical isolates from the PC I grouped closely with LS18 and LS21, isolated during the 1983 milk outbreak in Massachusetts [45], and branched away from all of the cantaloupe outbreaks strains from the PCs III and IV. This result suggested that, while there are three PCs of *L. monocytogenes* isolates serotype 1/2a implicated in this 2011 cantaloupe outbreak, the PC I strains were genetically distant from the other two PCs, which in turn are closely related, confirming the PFGE results. Further comparison of genetic contents in the PCs III and IV with those from PC I revealed that approximately 3% of probe-sets were uniquely present in each group (Tables S1 and S2). Figures 5A and 5B revealed the distribution of approximately 600 probe-sets uniquely present in PC I or PC II and III, respectively, into their different functional groups. Out of these probe-sets, the majority encoded hypothetical proteins followed by a large number of intergenic sequences, perhaps containing regulatory sequences or sRNAs. While the role of hypothetical proteins in virulence and eco-physiology of *Listeria* has not been established, the role of a few non-coding sRNA in pathogenesis and stress response has been clearly demonstrated [46], [47]

**Figure 6:**
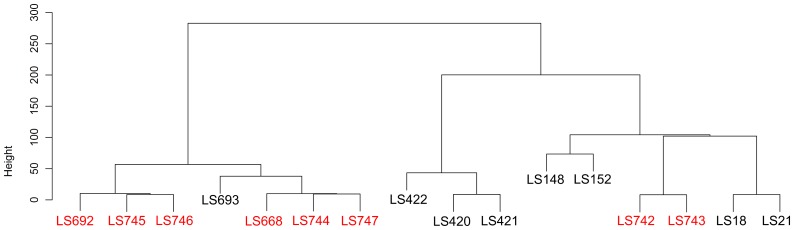
Hierarchical clustering based on the Robust Multi Array (RMA) analysis. Strains highlighted in red indicated the serotype 1/2a strains from the 2011 Cantaloupe outbreak.

**Figure 7:**
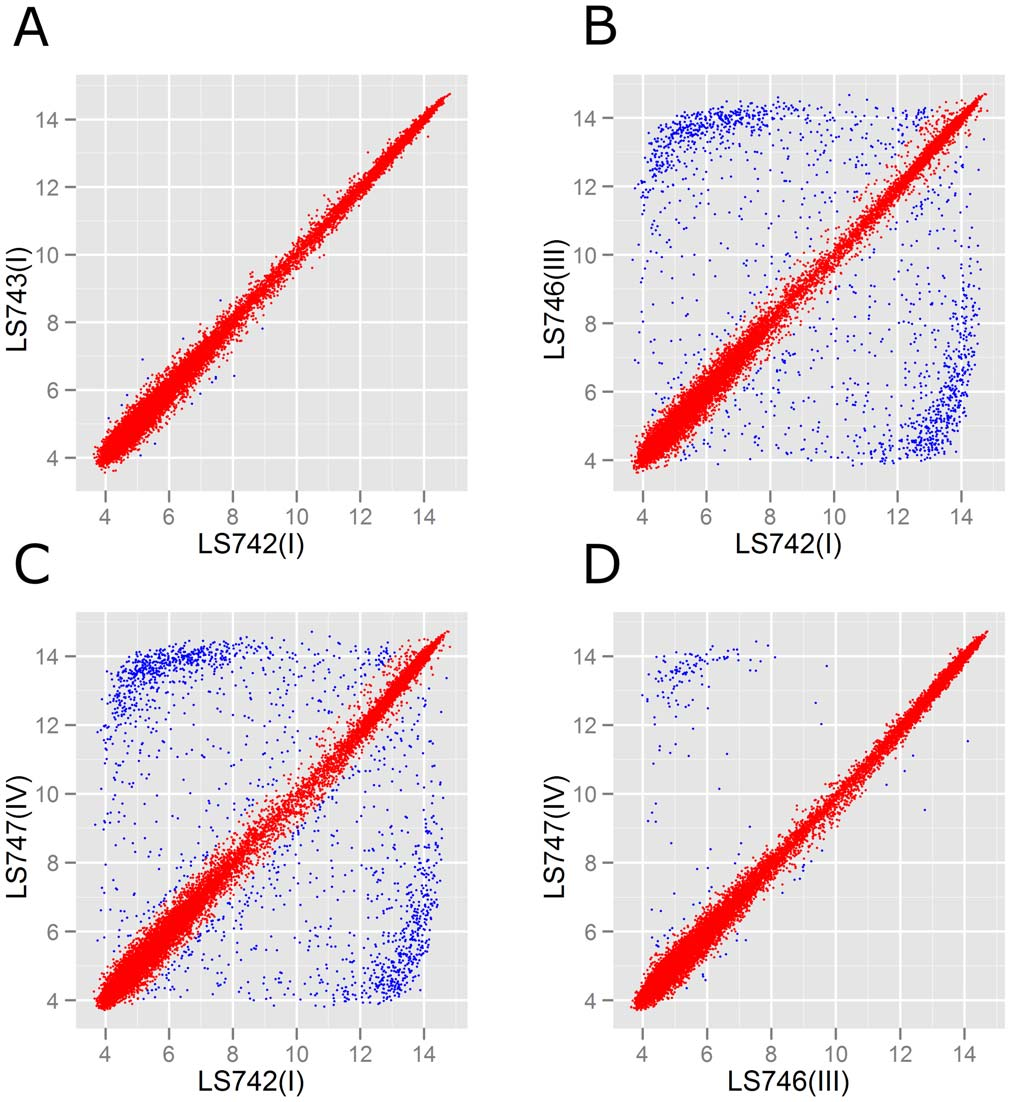
Comparison of the RMA summarized probe-set intensities. (A) pattern combination I isolates - LS742 and LS743; (B) pattern combination I (LS742) and pattern combination III (LS746); (C) pattern combination I (LS742) and pattern combination IV (LS747); (D) pattern combination III (LS746) and pattern combination IV(LS747). Red dots indicate RMA summarized probe-set intensity differences of less than or equal to 2-fold between two strains. Blue dots indicate RMA summarized probe-set intensity differences of more than 2-fold between the two strains.

To further analyze the genetic variability of the serotype 1/2a isolates, Robust Multi Array (RMA) algorithm [36], [37], which assesses individual probe-set intensity without normalizing the intensity data with mismatched-probe information, was also used. The hierarchical clustering, based on the summarized probe-set intensity among the cantaloupe outbreak strains, unmatched strain (LS693) and other outbreak strains (Fig. 6), was consistent with the MAS5.0 analysis (Fig. 4). This finding confirmed the genetic relatedness among these cantaloupe outbreak strains. Again, the PC I isolates (LS742 and LS743) appeared to be much closer to LS18 and LS21 than the other cantaloupe outbreak isolates. The summarized probe-set intensity of LS742 and LS743 also confirmed the closer relationship between these two strains (Fig. 7A). Comparison of the summarized probe-set intensity, as shown in the scatter plots between LS742 (PC I) and LS746 (PC III) and LS742 and LS747 (PC IV), indicated that there were approximately 8.2% and 9.2% of probe-sets with more than a two-fold difference between the summarized probe-set intensities from PC I and PC III and between PC I and PC IV strain, respectively (Figures 7B and 7C). These results again confirmed our earlier assertion that the serotype 1/2a strains from the cantaloupe outbreak isolates form two distinct genovars representing PC I in one and PCs III and IV in the other.

**Figure 8:**
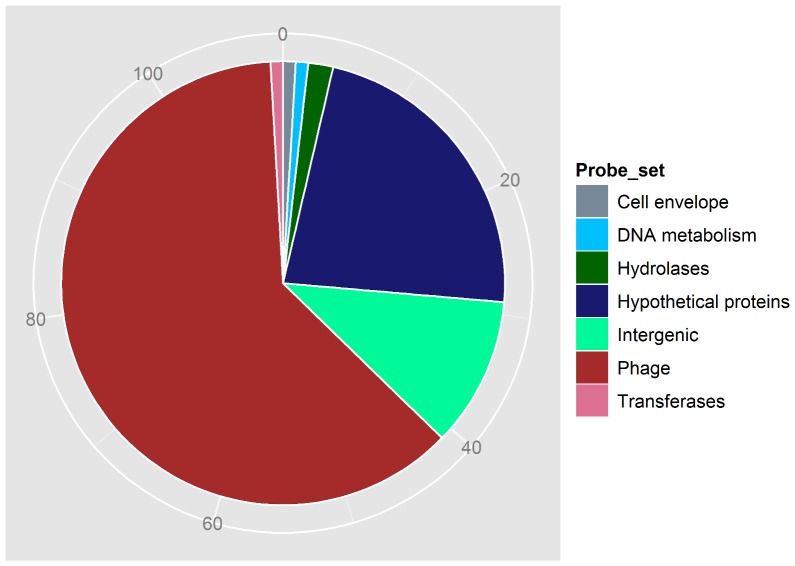
Diagrammatic representation of the distribution of unique probe-sets of the serotype 1/2a, PFGE pattern combination IV 2011 and absent in the PFGE pattern combination I and III strains based on their functions. The numbers of probe-sets were indicated in the periphery of the pie-chart.

The majority of the serotype 1/2a isolates from the cantaloupe outbreak strains analyzed here belong to the PCs III and IV. Comparison of gene contents from all of these six isolates based on MAS5.0 analysis showed that 7,643 probe-sets were present and 10,415 probe-sets were absent, which encompassed 97% of conserved probe-sets. This result indicated that isolates from the PCs III and IV are more closely related to each other than to the PC I strains and could be considered variants of the same strain. There were three unique probe-sets present in the isolates from PC III, including two hypothetical protein genes and one intergenic region. On the other hand, the PC IV strains contained an additional 110 unique probe-sets (Table S3, Fig. 8) that were absent in those from the PC III isolates. The RMA plot comparing summarized probe-set intensity from LS746 (PC III) and LS747 (PC IV) also revealed that only 0.75% of probe-sets have a higher summarized probe-set intensity in the PC IV than PC III strains indicating that these probes are unique (Fig. 7D). These results were consistent with those found using MAS5.0, confirming the relationship between PCs III and IV. Horizontal gene transfer may have played a significant role in separating these strains into two clusters as more than 70% of the additional probe-sets uniquely present in the PC IV strains are homologous to phage-related genes (Table S3). This is supported by the observations made by Chen and Novick that *L. monocytogenes* serotype 1/2a strains were most amenable to phage transduction compared to other serotypes, including 1/2b and 4b [48]. The role of the phage genome in the evolution of bacterial fitness, whether it is in the environment, food processing plants or humans, has generated serious discussion. Recently, Verghese et al. have shown that the phage genes integrated near *comK* could provide an added advantage for *Listeria* to survive in selective environments [49]. This could be achieved by directly affecting genome topography, thereby altering the transcription pattern or by providing a facile site for recombinogenic activity leading to widening of the gene pool and the potential for increased fitness. A recent study by Orsi et al. [50] showed that the major difference between isolates obtained from a 1988 sporadic listeriosis case and the 2000 listeriosis outbreak is the gain of a plasmid and sequence variability in a 42 Kb prophage genome integrated at the *comK* site. Thus, the increased ability to accept, maintain, and recombine/mutate phage gene sequences in serotype 1/2a PC IV strains could potentially contribute to the evolution of a more fit *L. monocytogenes* genovar. This raises an important question about the potential risk associated with the using of bacteriophages to control *Listeria* in foods. However, experimental evidence is required to determine whether isolates harboring the phage have a selective advantage relative to those that do not carry the phage and under what conditions the phage confers such an advantage.

### Cantaloupe outbreak isolates of serotype 1/2b

**Figure 9:**
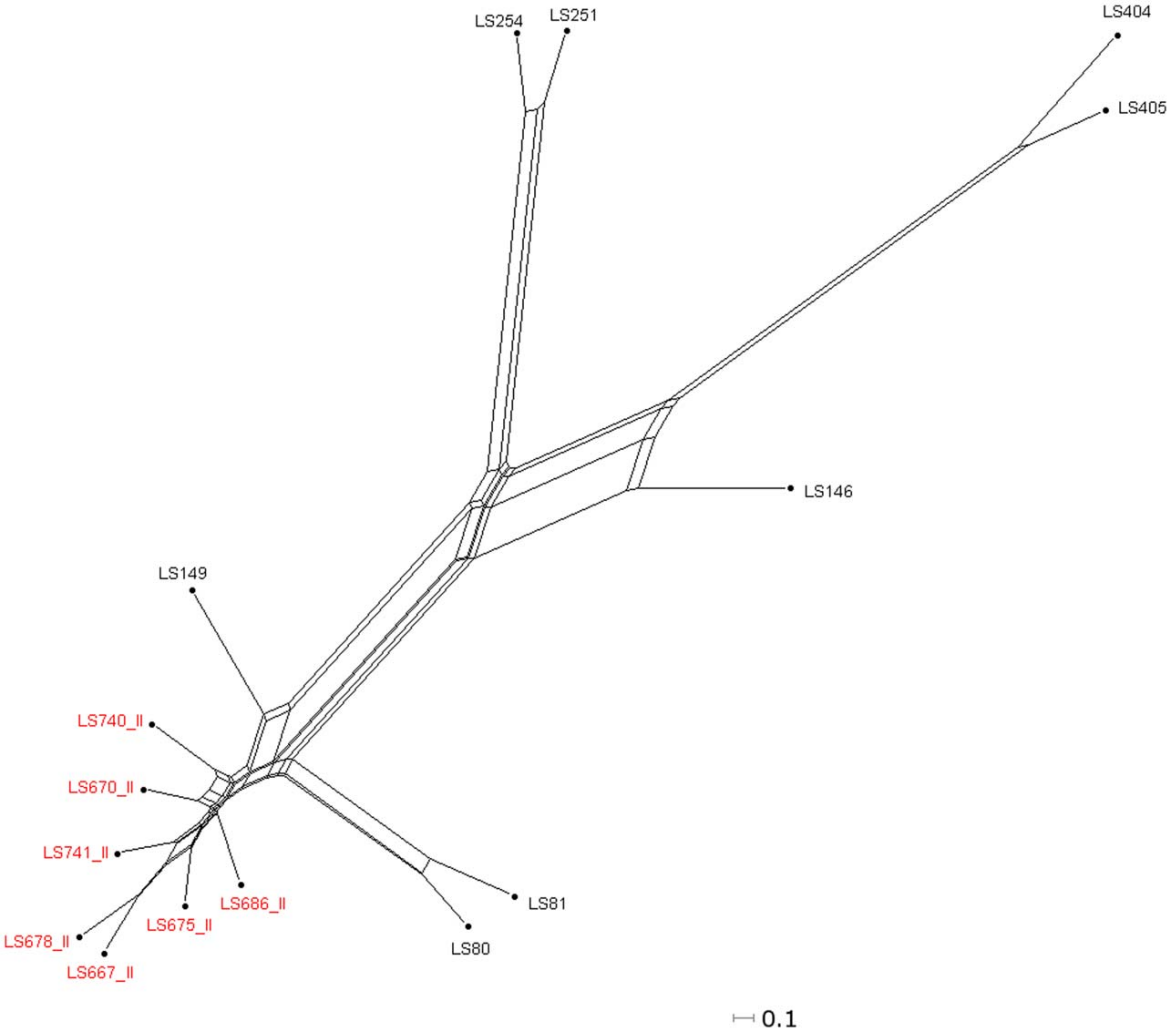
Relatedness analysis of the compatible parsimony informative genes from the 7 cantaloupe outbreak and 8 unrelated *L. monocytogenes* strains of serotype 1/2b [**28**]. The tree was generated from the concatenated gene contents using neighbor joining with the uncorrected p distance. Strains highlighted in red indicated the cantaloupe outbreak and unmatched strains. Scale bar represents number of gene differences (present or absent) per gene site.

**Figure 10:**
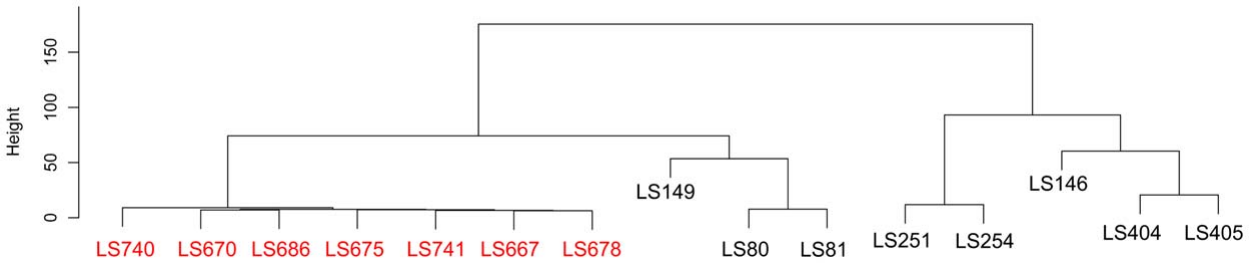
Hierarchical clustering based on the Robust Multi Array (RMA) analysis. Strains highlighted in red indicated the serotype 1/2b strains from the 2011 Cantaloupe outbreak.

The genetic relationship of the cantaloupe outbreak serotype 1/2b strains was examined based on the gene contents information obtained from the microarray analysis. The results indicated that 87.9% of total probe-sets were conserved among 1/2b isolates included in this study. Although the isolates from the cantaloupe outbreak are clustered together (Fig. 9), the genetic relatedness among these strains appears to be more dispersed than those found in the serotype 1/2a isolates. In addition, only three probe-sets, LMBG_01389_at, LMHCC_2728_s_at and lmo1556_s_at, were found to be uniquely present in all of the serotype 1/2b cantaloupe outbreak strains. Interestingly, the cantaloupe outbreak isolates, which caused Inv listeriosis, clustered closely to unrelated strains that previously caused sporadic Inv listeriosis (LS80, LS81 and LS149) and branched away from most of the FG outbreak strains (LS251, LS254, LS404 and LS405). Comparison of the gene contents between the FG and Inv groups revealed that approximately 0.7% of probe-sets are uniquely present in each group. RMA analysis of the serotype 1/2b cantaloupe outbreak isolates and the unrelated strains was consistent with the MAS5.0 analysis that the cantaloupe outbreak strains clustered together and formed a genovar closely related to the sporadic Inv strains (LS80 and LS81, Fig. 10).

After these analyses were completed, a state public health laboratory retrospectively identified a strain with a PFGE pattern combination different from all other PC in the national data base. This strain was detected on a sample of cantaloupe and only a single case of human illness was reported during the outbreak.

To summarize, a combination of serotyping, PFGE and DNA microarray hybridization analyses were employed to characterize the genomic footprints of the *L. monocytogenes* isolates that caused the large, multistate outbreak of listeriosis in 2011. The outbreak was unique in the sense that it was the first documentation of a listeriosis outbreak associated with consumption of contaminated cantaloupe in the United States and was caused by several distinct serotypes and genotypes of *L. monocytogenes*. The increasing discriminatory powers of the three analytical tools applied in this study underscores the utility of multiphasic approach in revealing the complex polyclonal nature of this outbreak. Our microarray analysis showed that the 1/2a and 1/2b strains associated with this outbreak are distinctly different from the previously reported 1/2a and 1/2b strains associated with both Inv and FG listeriosis outbreaks and sporadic cases. The pan-genomic analysis of these strains provided a powerful tool for exploring the genomic footprints of these organisms and underlined the possibility that phage genomes may be an important driving force behind the evolution of some strains of *L. monocytogenes*.

## Supporting Information

Table S1 **Probe-sets uniquely present in PC I serotype 1/2a.**
(DOCX)

Table S2 **Probe-sets uniquely present in PC III and IV strains serotype 1/2a.**
(DOCX)

Table S3 **Probe-sets uniquely present in PC IV serotype 1/2a.1.**
(DOCX)
