# Supplementary material for: Genomic Characterization of Listeria monocytogenes Strains Involved in a Multistate Listeriosis Outbreak Associated with Cantaloupe in US
Source: PLoS One. 2012 Jul 31;7(7):e42448. doi: 10.1371/journal.pone.0042448 (PMC3409164; doi:10.1371/journal.pone.0042448)
Supplement: Table S1 — Probe-sets uniquely present in PC I serotype 1/2a. (DOCX) [file pone.0042448.s001.docx]

**Supporting Information Table S1**: **Probe-sets uniquely present in PC I serotype 1/2a**

| **Probe ID** | **Annotation** |
| --- | --- |
| AARI_0068_s_at | 99% similar to LMHCC_2375 |
| AARI_0069_s_at | NK |
| AARI_0075_s_at | NK |
| AARI_0221_x_at | 99% similar to lmo0897 |
| AARI_0239_at | 98% similar to lmo0901 |
| AARI_0244_s_at | 98% similar to LMHCC_2380 |
| AARI_0309_s_at | 98% similar to lmo0723 |
| AARI_0318_s_at | NK |
| AARI_0325_s_at | 98% similar to LMHCC_2378 |
| AARI_0328_s_at | 99% similar to LMHCC_0054 |
| AARI_0334_s_at | NK |
| AARI_0337_x_at | NK |
| AARI_0338_x_at | NK |
| AARI_0346_at | NK |
| AARI_0431_s_at | 99% similar to lmo2760 |
| AARI_0449_s_at | 100% similar to lmo2446 |
| AARI_0469_s_at | NK |
| AARI_0479_s_at | 99% similar to lmo1838 |
| AARI_0502_s_at | NK |
| AARI_0510_x_at | 98% similar to lmo2470 |
| AARI_0553_s_at | 99% similar to LMHCC_0760 |
| AARI_0615_x_at | 100% similar to LMOf2365_0491 |
| AARI_0622_s_at | 99% similar to lmo1811 |
| AARI_0629_x_at | NK |
| AARI_0692_x_at | 99% similar to lmo1479 |
| AARK_0198_s_at | 99% similar to LMOf2365_0095 |
| AARK_1185_s_at | 100% similar to LMOf2365_0510 |
| AARK_1755_s_at | 100% similar to LMOf2365_1702 |
| AARK_1862_s_at | NK |
| AARK_1905_s_at | 100% similar to LMOf2365_1498 |
| AARL_0186_x_at | NK |
| AARL_0257_x_at | 98% similar to LMHCC_2123 |
| AARL_0284_s_at | NK |
| AARL_0295_x_at | NK |
| AARL_0356_x_at | NK |
| AARL_0602_at | NK |
| AARL_0669_at | NK |
| AARL_0669_x_at | NK |
| AARL_0702_x_at | NK |
| AARL_0704_x_at | NK |
| AARL_0707_x_at | NK |
| AARL_0808_at | 98% similar to LMHCC_0399 |
| AARL_0816_at | NK |
| AARL_0868_s_at | 99% similar to lmo0678 |
| AARM_0092_s_at | NK |
| AARM_0100_at | NK |
| AARM_0103_x_at | NK |
| AARM_0295_s_at | 99% similar to lmo2138 |
| AARM_0410_at | NK |
| AARM_0472_at | 98% similar to lmo1927 |
| AARM_0539_x_at | 98% similar to lmo2693 |
| AARM_0824_s_at | 99% similar to lmo1728 |
| AARM_0844_s_at | NK |
| AARM_0875_at | NK |
| AARM_0875_x_at | NK |
| AARM_0959_at | 98% similar to lmo1675 |
| AARM_0988_s_at | 98% similar to lmo0489 |
| AARM_1125_s_at | 100% similar to lmo1838 |
| AARM_1323_s_at | NK |
| AARM_1327_x_at | NK |
| AARM_1422_s_at | 99% similar to LMHCC_1448 |
| AARM_1482_at | NK |
| AARM_1557_s_at | NK |
| AARM_1630_s_at | NK |
| AARM_1652_at | NK |
| AARM_1658_x_at | 98% similar to lmo1987 |
| AARM_1665_at | 100% similar to lmo1360 |
| AARM_1775_s_at | NK |
| AARO_0227_s_at | NK |
| AARO_0227_x_at | NK |
| AARO_1102_s_at | 100% similar to LMOf2365_1391 |
| AARO_1143_s_at | 99% similar to LMOf2365_0063 |
| AARO_1671_s_at | 100% similar to LMOf2365_2417 |
| AARO_1687_x_at | 100% similar to LMOf2365_1628 |
| AARO_1738_at | NK |
| AARO_1857_s_at | NK |
| AARY_0088_s_at | 99% similar to lmo2121 |
| AARY_0114_s_at | 100% similar to lmo1821 |
| AARY_0115_s_at | 99% similar to lmo1820 |
| AARY_0176_s_at | 99% similar to lmo1913 |
| AARY_0201_s_at | 99% similar to lmo1759 |
| AARY_0549_s_at | 100% similar to lmo0199 |
| AARY_0684_s_at | 99% similar to lmo0401 |
| AARY_0715_s_at | 99% similar to lmo2172 |
| AARY_0854_x_at | 100% similar to lmo2766 |
| AARY_0860_s_at | 100% similar to lmo1165 |
| AARY_0864_s_at | 99% similar to lmo0327 |
| AARY_0961_x_at | 99% similar to lmo1135 |
| AARY_0964_s_at | 100% similar to lmo2591 |
| AARY_1136_s_at | 99% similar to lmo2561 |
| AARY_1157_s_at | 100% similar to lmo0841 |
| AARY_1177_x_at | 100% similar to lmo1431 |
| AARY_1222_s_at | 100% similar to lmo1360 |
| AARY_1354_x_at | 100% similar to lmo2023 |
| AARY_1408_s_at | 100% similar to lmo1764 |
| AARY_1409_s_at | 100% similar to lmo1764 |
| AARY_1417_at | 100% similar to lmo1286 |
| AARY_1545_s_at | 100% similar to lmo1559 |
| AARY_1570_s_at | 100% similar to lmo1660 |
| IGLm4b_00274_x_at | intergenic region |
| IGLm4b_00397_at | intergenic region |
| IGLm4b_00397_x_at | intergenic region |
| IGLm4b_00488_x_at | intergenic region |
| IGLm4b_01128_at | intergenic region |
| IGLm4b_01489_x_at | intergenic region |
| IGLm4b_02138_x_at | intergenic region |
| IGLm4b_02207_x_at | intergenic region |
| IGLm4b_02290_x_at | intergenic region |
| IGLMHCC_0098_s_at | intergenic region |
| IGLMHCC_0287_at | intergenic region |
| IGLMHCC_0300_at | intergenic region |
| IGLMHCC_0401_at | intergenic region |
| IGLMHCC_0534_x_at | intergenic region |
| IGLMHCC_0575_s_at | intergenic region |
| IGLMHCC_0583_x_at | intergenic region |
| IGLMHCC_1058_at | intergenic region |
| IGLMHCC_1092_at | intergenic region |
| IGLMHCC_1114_s_at | intergenic region |
| IGLMHCC_1316_at | intergenic region |
| IGLMHCC_1318_s_at | intergenic region |
| IGLMHCC_1333_at | intergenic region |
| IGLMHCC_1333_x_at | intergenic region |
| IGLMHCC_1387_at | intergenic region |
| IGLMHCC_1387_x_at | intergenic region |
| IGLMHCC_1434_at | intergenic region |
| IGLMHCC_2069_x_at | intergenic region |
| IGLMHCC_2079_at | intergenic region |
| IGLMHCC_2165_x_at | intergenic region |
| IGLMHCC_2168_x_at | intergenic region |
| IGLMHCC_2169_at | intergenic region |
| IGLMHCC_2169_x_at | intergenic region |
| IGLMHCC_2181_at | intergenic region |
| IGLMHCC_2182_at | intergenic region |
| IGLMHCC_2230_at | intergenic region |
| IGLMHCC_2385_at | intergenic region |
| IGLMHCC_2419_x_at | intergenic region |
| IGLMHCC_2862_at | intergenic region |
| IGLMHCC_2862_x_at | intergenic region |
| IGlmo0081_at | intergenic region |
| IGlmo0082_at | intergenic region |
| IGlmo0115_at | intergenic region |
| IGlmo0142_at | intergenic region |
| IGlmo0143_at | intergenic region |
| IGlmo0144_at | intergenic region |
| IGlmo0145_at | intergenic region |
| IGlmo0145_x_at | intergenic region |
| IGlmo0146_s_at | intergenic region |
| IGlmo0146_x_at | intergenic region |
| IGlmo0175_at | intergenic region |
| IGlmo0281_at | intergenic region |
| IGlmo0293_at | intergenic region |
| IGlmo0293_x_at | intergenic region |
| IGlmo0295_s_at | intergenic region |
| IGlmo0296_at | intergenic region |
| IGlmo0296_s_at | intergenic region |
| IGlmo0296_x_at | intergenic region |
| IGlmo0324_x_at | intergenic region |
| IGlmo0334_at | intergenic region |
| IGlmo0339_at | intergenic region |
| IGlmo0424_at | intergenic region |
| IGlmo0452_at | intergenic region |
| IGlmo0452_x_at | intergenic region |
| IGlmo0459_at | intergenic region |
| IGlmo0460_at | intergenic region |
| IGlmo0464_x_at | intergenic region |
| IGlmo0662_x_at | intergenic region |
| IGlmo0792_x_at | intergenic region |
| IGlmo0866_at | intergenic region |
| IGlmo0900_at | intergenic region |
| IGlmo0981_x_at | intergenic region |
| IGlmo1014_at | intergenic region |
| IGlmo1014_x_at | intergenic region |
| IGlmo1190_at | intergenic region |
| IGlmo1479_x_at | intergenic region |
| IGlmo1571_at | intergenic region |
| IGlmo1572_at | intergenic region |
| IGlmo1604_at | intergenic region |
| IGlmo1648_at | intergenic region |
| IGlmo1649_at | intergenic region |
| IGlmo1659_at | intergenic region |
| IGlmo1660_at | intergenic region |
| IGlmo1762_x_at | intergenic region |
| IGlmo2034_at | intergenic region |
| IGlmo2256_at | intergenic region |
| IGlmo2272_at | intergenic region |
| IGlmo2310_at | intergenic region |
| IGlmo2444_at | intergenic region |
| IGlmo2500_at | intergenic region |
| IGlmo2500_x_at | intergenic region |
| IGlmo2538_at | intergenic region |
| IGlmo2538_x_at | intergenic region |
| IGlmo2560_at | intergenic region |
| IGlmo2760_x_at | intergenic region |
| IGLMOf2365_0151_s_at | intergenic region |
| IGLMOf2365_0156_s_at | intergenic region |
| IGLMOf2365_0161_at | intergenic region |
| IGLMOf2365_0161_s_at | intergenic region |
| IGLMOf2365_0163_x_at | intergenic region |
| IGLMOf2365_0188_x_at | intergenic region |
| IGLMOf2365_0266_s_at | intergenic region |
| IGLMOf2365_0267_s_at | intergenic region |
| IGLMOf2365_0272_s_at | intergenic region |
| IGLMOf2365_0274_s_at | intergenic region |
| IGLMOf2365_0285_s_at | intergenic region |
| IGLMOf2365_0330_s_at | intergenic region |
| IGLMOf2365_0372_s_at | intergenic region |
| IGLMOf2365_0481_at | intergenic region |
| IGLMOf2365_0482_s_at | intergenic region |
| IGLMOf2365_0493_x_at | intergenic region |
| IGLMOf2365_0498_x_at | intergenic region |
| IGLMOf2365_0505_s_at | intergenic region |
| IGLMOf2365_0696_x_at | intergenic region |
| IGLMOf2365_0860_x_at | intergenic region |
| IGLMOf2365_0921_x_at | intergenic region |
| IGLMOf2365_1162_x_at | intergenic region |
| IGLMOf2365_1257_s_at | intergenic region |
| IGLMOf2365_1275_at | intergenic region |
| IGLMOf2365_1275_x_at | intergenic region |
| IGLMOf2365_1365_at | intergenic region |
| IGLMOf2365_1498_x_at | intergenic region |
| IGLMOf2365_1573_x_at | intergenic region |
| IGLMOf2365_1621_x_at | intergenic region |
| IGLMOf2365_1733_x_at | intergenic region |
| IGLMOf2365_2176_x_at | intergenic region |
| IGLMOf2365_2177_at | intergenic region |
| IGLMOf2365_2177_x_at | intergenic region |
| IGLMOf2365_2253_x_at | intergenic region |
| IGLMOf2365_2366_at | intergenic region |
| IGLMOf2365_2537_x_at | intergenic region |
| IGLMOf2365_2796_x_at | intergenic region |
| IGLMOf2365_2797_at | intergenic region |
| IGLMOf2365_2797_x_at | intergenic region |
| Lm4b_01128_at | GI=225876187 |
| Lm4b_01270_s_at | proB gamma-glutamyl kinase/GI=225876325 |
| Lm4b_01815_s_at | Putative peptidoglycan bound protein (LPXTG motif)/GI=225876864 |
| Lm4b_02041_s_at | Hypothetical protein of unknown function/GI=225877088 |
| Lm4b_02459_x_at | Putative CsbA protein/GI=225877500 |
| LMBG_01686_at | phage protein |
| LMBG_01686_x_at | phage protein |
| LMBG_02156_x_at | thiaminephosphate pyrophosphorylase/Pfam=PF02581.9 |
| LMFG_00724_s_at | D-alanine-D-alanine ligase |
| LMFG_02515_at | predicted protein |
| LMFG_02674_at | predicted protein |
| LMFG_02675_at | conserved hypothetical protein |
| LMFG_02675_x_at | conserved hypothetical protein |
| LMFG_02953_s_at | conserved hypothetical protein |
| LMFG_03116_s_at | predicted protein/Pfam=PF00746.13 |
| LMFG_03220_x_at | predicted protein |
| LMFG_03225_s_at | conserved hypothetical protein |
| LMFG_03235_s_at | predicted protein |
| LMHCC_0008_s_at | alkaline phosphatase synthesis transcriptional regulatory proteinphoP/GI=217332580 |
| LMHCC_0010_at | ABC transporter, permease protein/GI=217332582 |
| LMHCC_0098_s_at | membrane protein, putative/GI=217332666 |
| LMHCC_0260_s_at | L-cystine import ATP-binding protein TcyN/GI=217332826 |
| LMHCC_0348_s_at | oligopeptide transport system permease protein OppC/GI=217332914 |
| LMHCC_0401_at | mannose-6-phosphate isomerase, class I, putative/GI=217332966 |
| LMHCC_0401_s_at | mannose-6-phosphate isomerase, class I, putative/GI=217332966 |
| LMHCC_0537_x_at | nadB L-aspartate oxidase/GI=217333100 |
| LMHCC_0832_x_at | glutamate synthase (NADPH) small chain/GI=217333390 |
| LMHCC_0833_s_at | ABC transporter permease protein/GI=217333391 |
| LMHCC_0834_s_at | ABC transporter, permease protein/GI=217333392 |
| LMHCC_1056_s_at | cysteine desulfurase/GI=217333610 |
| LMHCC_1057_x_at | trmU tRNA (5-methylaminomethyl-2-thiouridylate)-methyltransferase/GI=217333611 |
| LMHCC_1091_s_at | lepA GTP-binding protein LepA/GI=217333645 |
| LMHCC_1117_s_at | S-adenosylmethionine/GI=217333671 |
| LMHCC_1156_s_at | conserved hypothetical protein/GI=217333710 |
| LMHCC_1199_s_at | 2-oxoisovalerate dehydrogenase subunit alpha (branched-chain alpha-keto acid dehydrogenase e1 component alpha chain)/GI=217333752 |
| LMHCC_1223_s_at | gcvT glycine cleavage system T protein/GI=217333776 |
| LMHCC_1315_s_at | hydrolase, alpha-beta fold family/GI=217333866 |
| LMHCC_1333_x_at | conserved hypothetical protein/GI=217333884 |
| LMHCC_1489_s_at | propanediol utilization protein PduM/GI=217334039 |
| LMHCC_1774_s_at | small multidrug resistance protein/GI=217334321 |
| LMHCC_2150_x_at | MutTnudix family protein/GI=217334694 |
| LMHCC_2164_x_at | transposase OrfA, IS3 family, putative/GI=217334708 |
| LMHCC_2167_s_at | conserved hypothetical protein/GI=217334711 |
| LMHCC_2182_s_at | PBP 5 synthesis repressor/GI=217334726 |
| LMHCC_2211_x_at | pts system fructose-specific eiibbc component (eiibbc-fru)/GI=217334755 |
| LMHCC_2333_s_at | PRDPTS system IIA 2 domain regulatory protein/GI=217334876 |
| LMHCC_2497_s_at | hypothetical protein/GI=217335038 |
| LMHCC_2520_s_at | ABC transporter, ATP-bindingpermease protein/GI=217335061 |
| LMHCC_2600_s_at | glyoxylate reductase (Glycolate reductase)/GI=217335141 |
| LMHCC_2600_x_at | glyoxylate reductase (Glycolate reductase)/GI=217335141 |
| LMHCC_2771_s_at | hydrolase, CocENonD family/GI=217335311 |
| LMHCC_2803_s_at | HAD-superfamily hydrolase, subfamily IA, variant 1/GI=217335342 |
| LMHCC_2836_x_at | phosphosugar-binding transcriptional regulator, RpiR family/GI=217335374 |
| LMHCC_2859_at | hydrolase, alphabeta fold family/GI=217335396 |
| LMHCC_2859_x_at | hydrolase, alphabeta fold family/GI=217335396 |
| LMHCC_2860_x_at | ImpBMucBSamB family protein/GI=217335397 |
| LMHCC_2861_s_at | conserved hypothetical protein/GI=217335398 |
| LMHCC_2889_s_at | conserved hypothetical protein/GI=217335426 |
| LMHCC_3006_s_at | lipoprotein, putative/GI=217335543 |
| LMHG_00214_at | ABC transporter/Pfam=PF01497.10 |
| LMHG_00237_at | hisA/Pfam=PF00977.13 |
| LMHG_00512_at | conserved hypothetical protein/Pfam=PF07006.3 |
| LMHG_00512_x_at | conserved hypothetical protein/Pfam=PF07006.3 |
| LMHG_00893_s_at | ImpBMucBSamB family protein/Pfam=PF00817.12 |
| LMHG_00894_x_at | hydrolase/Pfam=PF00561.12 |
| LMHG_00906_s_at | hypothetical protein |
| LMHG_00908_at | Nacetylmuramoyl-L-alanine amidase/Pfam=PF01832.12 |
| LMHG_00908_s_at | Nacetylmuramoyl-L-alanine amidase/Pfam=PF01832.12 |
| LMHG_00910_x_at | thymidylate kinase/Pfam=PF02223.9 |
| LMHG_01289_at | alsS/Pfam=PF02776.10 |
| LMHG_01437_x_at | hydrolase/Pfam=PF00561.12 |
| LMHG_01678_x_at | Disomer specific 2hydroxyacid dehydrogenase/Pfam=PF02826.11 |
| LMHG_01684_at | CBS domaincontaining protein/Pfam=PF07085.4 |
| LMHG_01923_x_at | MutTnudix family protein/Pfam=PF00293.20 |
| LMHG_02484_at | conserved hypothetical protein/Pfam=PF04055.13 |
| LMHG_02678_s_at | propanediol utilization protein PduM |
| LMHG_03094_at | conserved hypothetical protein |
| LMHG_03149_at | PTS system protein |
| LMHG_03157_x_at | peptidoglycan binding protein/Pfam=PF00746.13 |
| LMIG_00006_at | crisprassociated protein/Pfam=PF09711.2 |
| LMIG_00007_at | crisprassociated protein cas2 |
| LMIG_00333_s_at | DHH family protein/Pfam=PF02272.11 |
| LMIG_00411_s_at | EAL domaincontaining protein/Pfam=PF00563.12 |
| LMIG_00413_x_at | conserved hypothetical protein/Pfam=PF06902.3 |
| LMIG_00630_s_at | acetolactate synthase 3 regulatory subunit/Pfam=PF01842.17 |
| LMIG_00662_at | alphaDmannosidase/Pfam=PF09261.3 |
| LMIG_01005_s_at | septum sitedetermining protein MinC/Pfam=PF03775.8 |
| LMIG_01364_s_at | trehalose6phosphate hydrolase/Pfam=PF00128.16 |
| LMIG_01517_at | glycosyl hydrolase/Pfam=PF03422.7 |
| LMIG_01913_x_at | conserved hypothetical protein/Pfam=PF00356.13 |
| LMIG_01920_s_at | phosphoglucomutasephosphomannomutase/Pfam=PF02879.8 |
| LMIG_01940_s_at | protoporphyrinogen oxidase/Pfam=PF01593.16 |
| LMIG_01965_s_at | phosphoglycerate mutase/Pfam=PF00300.14 |
| LMIG_02175_s_at | conserved hypothetical protein |
| LMIG_02249_x_at | conserved hypothetical protein/Pfam=PF05675.4 |
| LMIG_02280_at | GTPbinding protein TypA/Pfam=PF08477.5 |
| LMIG_02564_s_at | betaglucosidase/Pfam=PF01915.14 |
| LMIG_02570_s_at | transcriptional regulator GltC/Pfam=PF03466.12 |
| LMIG_02662_at | NADH:flavin oxidoreductase/Pfam=PF00724.12 |
| LMIG_02662_x_at | NADH:flavin oxidoreductase/Pfam=PF00724.12 |
| LMIG_02677_s_at | alcohol dehydrogenase/Pfam=PF08240.4 |
| LMIG_02826_s_at | conserved hypothetical protein/Pfam=PF01841.11 |
| LMIG_02843_x_at | conserved hypothetical protein/Pfam=PF01527.12 |
| LMIG_02887_s_at | predicted protein |
| LMIG_02909_s_at | predicted protein |
| LMIG_02910_s_at | predicted protein |
| LMIG_02939_at | predicted protein |
| LMIG_02947_s_at | predicted protein |
| LMIG_02948_s_at | conserved hypothetical protein |
| LMJG_02806_s_at | predicted protein |
| LMKG_00453_s_at | predicted protein |
| LMKG_01576_at | predicted protein |
| LMKG_01623_x_at | predicted protein |
| LMKG_01740_at | predicted protein |
| LMKG_01740_x_at | predicted protein |
| LMKG_02495_x_at | conserved hypothetical protein |
| LMLG_00020_x_at | conserved hypothetical protein |
| LMLG_00205_at | major facilitator family transporter/Pfam=PF07690.8 |
| LMLG_00210_x_at | conserved hypothetical protein |
| LMLG_00291_at | glycosyl hydrolase/Pfam=PF01074.14 |
| LMLG_00315_at | conserved hypothetical protein/Pfam=PF00881.16 |
| LMLG_00377_x_at | pantetheinephosphate adenylyltransferase/Pfam=PF01467.18 |
| LMLG_00453_at | glucose6phosphate 1dehydrogenase/Pfam=PF02781.8 |
| LMLG_00524_at | glutamate dehydrogenase/Pfam=PF02812.10 |
| LMLG_00527_s_at | imidazoleglycerol phosphate synthase/Pfam=PF00977.13 |
| LMLG_00570_at | conserved hypothetical protein/Pfam=PF01554.10 |
| LMLG_00741_x_at | conserved hypothetical protein |
| LMLG_00754_s_at | transcriptional regulator |
| LMLG_00790_at | internalin D/Pfam=PF09479.2 |
| LMLG_00804_at | RTCB proteinlike protein/Pfam=PF01139.9 |
| LMLG_00804_x_at | RTCB proteinlike protein/Pfam=PF01139.9 |
| LMLG_00815_x_at | RNA polymerase sigma30 factor/Pfam=PF08281.4 |
| LMLG_00838_s_at | iron compound ABC transporter/Pfam=PF01032.10 |
| LMLG_00951_at | conserved hypothetical protein/Pfam=PF03466.12 |
| LMLG_00991_at | conserved hypothetical protein |
| LMLG_00991_x_at | conserved hypothetical protein |
| LMLG_00992_at | glutamate 5kinase/Pfam=PF00696.20 |
| LMLG_00993_at | gammaglutamyl phosphate reductase |
| LMLG_00994_x_at | hydrolase/Pfam=PF00561.12 |
| LMLG_00996_at | MutTnudix family protein/Pfam=PF00293.20 |
| LMLG_00996_x_at | MutTnudix family protein/Pfam=PF00293.20 |
| LMLG_00997_s_at | PTS system/Pfam=PF02378.10 |
| LMLG_00998_s_at | trehalose6phosphate hydrolase/Pfam=PF00128.16 |
| LMLG_01200_at | conserved hypothetical protein/Pfam=PF00425.10 |
| LMLG_01200_s_at | conserved hypothetical protein/Pfam=PF00425.10 |
| LMLG_01200_x_at | conserved hypothetical protein/Pfam=PF00425.10 |
| LMLG_01203_at | conserved hypothetical protein/Pfam=PF02574.8 |
| LMLG_01283_at | ribose 5phosphate isomerase B/Pfam=PF02502.10 |
| LMLG_01395_at | glutathione reductase/Pfam=PF07992.6 |
| LMLG_01402_s_at | succinatesemialdehyde dehydrogenase/Pfam=PF00171.14 |
| LMLG_01532_at | methionine aminopeptidase/Pfam=PF00557.16 |
| LMLG_01718_s_at | pyruvate formatelyase activating enzyme/Pfam=PF04055.13 |
| LMLG_01752_at | DHH subfamily protein/Pfam=PF02272.11 |
| LMLG_02001_at | sigma70 region 2 family protein/Pfam=PF08281.4 |
| LMLG_02001_x_at | sigma70 region 2 family protein/Pfam=PF08281.4 |
| LMLG_02040_at | thiaminepyrophosphaterequiring enzyme/Pfam=PF02775.13 |
| LMLG_02575_at | conserved hypothetical protein/Pfam=PF04095.8 |
| LMLG_02607_at | transcriptional regulator/Pfam=PF08279.4 |
| LMLG_02617_at | conserved hypothetical protein/Pfam=PF00232.10 |
| LMLG_02625_at | conserved hypothetical protein/Pfam=PF03239.6 |
| LMLG_02654_at | major facilitator family transporter/Pfam=PF07690.8 |
| LMLG_02829_at | propanediol utilization/Pfam=PF08841.2 |
| LMLG_02829_x_at | propanediol utilization/Pfam=PF08841.2 |
| LMLG_02853_x_at | glutamine amidotransferase/Pfam=PF07722.5 |
| lmo0079_s_at | GI=16409438 |
| lmo0080_at | GI=16409439 |
| lmo0081_at | GI=16409440 |
| lmo0082_at | GI=16409441 |
| lmo0140_s_at | GI=16409499 |
| lmo0143_s_at | GI=16409502 |
| lmo0145_s_at | GI=16409504 |
| lmo0241_s_at | GI=16409606 |
| lmo0242_at | GI=16409607 |
| lmo0242_x_at | GI=16409607 |
| lmo0256_x_at | GI=16409621 |
| lmo0281_s_at | GI=16409646 |
| lmo0294_s_at | GI=16409659 |
| lmo0317_s_at | GI=16409681 |
| lmo0335_x_at | GI=16409713 |
| lmo0336_x_at | GI=16409714 |
| lmo0337_s_at | GI=16409715 |
| lmo0338_s_at | GI=16409716 |
| lmo0342_s_at | GI=16409720 |
| lmo0369_s_at | GI=16409747 |
| lmo0427_x_at | GI=16409804 |
| lmo0458_s_at | GI=16409835 |
| lmo0461_s_at | GI=16409838 |
| lmo0463_s_at | GI=16409840 |
| lmo0487_s_at | GI=16409863 |
| lmo0488_s_at | GI=16409864 |
| lmo0636_at | GI=16410025 |
| lmo0645_s_at | GI=16410034 |
| lmo0861_at | GI=16410264 |
| lmo0861_x_at | GI=16410264 |
| lmo0865_at | GI=16410268 |
| lmo0915_at | GI=16410318 |
| lmo0923_s_at | GI=16410326 |
| lmo0947_s_at | GI=16410349 |
| lmo1135_s_at | GI=16410551 |
| lmo1168_at | AckA2 GI=16410584 |
| lmo1179_s_at | GI=16410595 |
| lmo1217_s_at | GI=16410633 |
| lmo1280_s_at | codY GI=16410696 |
| lmo1402_s_at | GI=16410831 |
| lmo1472_s_at | dnaJ heat shock protein DnaJ/GI=16410901 |
| lmo1573_s_at | accD GI=16411002 |
| lmo1604_s_at | GI=16411033 |
| lmo1647_s_at | GI=16411083 |
| lmo1648_at | GI=16411084 |
| lmo1659_at | GI=16411095 |
| lmo1685_s_at | gsaB glutamate-1-semialdehyde aminotransferase/GI=16411121 |
| lmo1735_s_at | gltC transcription activator of glutamate synthase operon GltC/GI=16411189 |
| lmo1765_s_at | purH Bifunctional phosphoribosylaminoimidazole carboxy formyl formyltransferase and inosine-monophosphate cyclohydrolase/GI=16411219 |
| lmo1837_s_at | pyrC GI=16411291 |
| lmo1913_s_at | GI=16411366 |
| lmo1917_at | pflA GI=16411370 |
| lmo1922_s_at | GI=16411375 |
| lmo1954_s_at | drm GI=16411407 |
| lmo2046_x_at | GI=16411516 |
| lmo2049_s_at | GI=16411519 |
| lmo2228_s_at | GI=16411698 |
| lmo2312_x_at | GI=16411782 |
| lmo2430_s_at | GI=16411918 |
| lmo2447_s_at | GI=16411935 |
| lmo2475_s_at | GI=16411963 |
| lmo2536_x_at | atpI GI=16412024 |
| lmo2537_s_at | GI=16412025 |
| lmo2558_at | ami "autolysin, amidase"/GI=16412046 |
| lmo2561_s_at | argS arginyl tRNA synthetase/GI=16412049 |
| lmo2593_at | GI=16412081 |
| lmo2634_s_at | GI=16412122 |
| lmo2662_s_at | GI=16412162 |
| lmo2683_at | GI=16412183 |
| lmo2689_x_at | GI=16412189 |
| lmo2695_at | GI=16412195 |
| lmo2695_x_at | GI=16412195 |
| lmo2721_s_at | GI=16412221 |
| lmo2748_at | GI=16412248 |
| lmo2760_at | GI=16412260 |
| lmo2812_s_at | GI=16412312 |
| LMOf2365_0019_s_at | qoxD quinol oxidase AA3, subunit IV/GI=46879506 |
| LMOf2365_0135_s_at | lmaB prophage LambdaLm01, antigen B/GI=46879621 |
| LMOf2365_0159_s_at | conserved hypothetical protein/GI=46879644 |
| LMOf2365_0160_s_at | hypothetical protein/GI=46879645 |
| LMOf2365_0162_s_at | conserved hypothetical protein/GI=46879647 |
| LMOf2365_0267_s_at | sugar ABC transporter, sugar-binding protein/GI=46879752 |
| LMOf2365_0269_s_at | sugar ABC transporter, permease protein/GI=46879754 |
| LMOf2365_0270_s_at | malL2 oligo-1,6-glucosidase/GI=46879755 |
| LMOf2365_0271_s_at | gtfA sucrose phosphorylase/GI=46879756 |
| LMOf2365_0272_s_at | hypothetical protein/GI=46879757 |
| LMOf2365_0273_s_at | hypothetical protein/GI=46879758 |
| LMOf2365_0284_s_at | peptidase, M20M25M40 family/GI=46879770 |
| LMOf2365_0304_s_at | metN D-methionine ABC transporter, ATP-binding protein/GI=46879790 |
| LMOf2365_0316_s_at | nitroreductase family protein/GI=46879802 |
| LMOf2365_0336_s_at | thiE thiamine-phosphate pyrophosphorylase/GI=46879821 |
| LMOf2365_0346_x_at | conserved hypothetical protein/GI=46879831 |
| LMOf2365_0354_s_at | conserved domain protein/GI=46879839 |
| LMOf2365_0355_at | conserved hypothetical protein/GI=46879840 |
| LMOf2365_0444_s_at | PTS system, fructose-specific, IIC component/GI=46879927 |
| LMOf2365_0474_s_at | putative ytfG protein/GI=46879956 |
| LMOf2365_0481_s_at | conserved domain protein/GI=46879963 |
| LMOf2365_0496_s_at | hypothetical protein/GI=46879978 |
| LMOf2365_0497_s_at | hypothetical protein/GI=46879979 |
| LMOf2365_0499_s_at | putative transposase OrfA, IS3 family/GI=46879981 |
| LMOf2365_0664_s_at | HAD-superfamily hydrolase, subfamily IA, variant 1/GI=46880146 |
| LMOf2365_0712_s_at | fliP flagellar biosynthesis protein FliP/GI=46880193 |
| LMOf2365_0733_s_at | putative flagellar hook protein FlgE/GI=46880214 |
| LMOf2365_0828_s_at | HD domain protein/GI=46880308 |
| LMOf2365_0854_s_at | ABC transporter, ATP-binding protein/GI=46880334 |
| LMOf2365_0929_s_at | phosphoglycerate mutase family protein/GI=46880409 |
| LMOf2365_0942_s_at | putative ABC transporter, permease protein/GI=46880422 |
| LMOf2365_1020_s_at | putative membrane protein/GI=46880498 |
| LMOf2365_1256_s_at | conserved hypothetical protein/GI=46880733 |
| LMOf2365_1257_s_at | hypothetical protein/GI=46880734 |
| LMOf2365_1269_s_at | conserved hypothetical protein/GI=46880746 |
| LMOf2365_1274_s_at | conserved hypothetical protein/GI=46880751 |
| LMOf2365_1348_s_at | pnp polyribonucleotide nucleotidyltransferase/GI=46880825 |
| LMOf2365_1363_s_at | general secretion pathway protein F/GI=46880840 |
| LMOf2365_1364_s_at | general secretion pathway protein E/GI=46880841 |
| LMOf2365_1376_s_at | nusB N utilization substance protein B/GI=46880853 |
| LMOf2365_1446_s_at | opuCB glycine betaineL-proline ABC transporter, permease protein/GI=46880923 |
| LMOf2365_1497_s_at | transcriptional regulator, MerR family/GI=46880975 |
| LMOf2365_1563_x_at | minD septum site-determining protein MinD/GI=46881041 |
| LMOf2365_1595_s_at | accD acetyl-CoA carboxylase, carboxyl transferase, beta subunit/GI=46881073 |
| LMOf2365_1617_s_at | GAF domain protein/GI=46881095 |
| LMOf2365_1624_s_at | putative aminopeptidase/GI=46881102 |
| LMOf2365_1761_s_at | alcohol dehydrogenase, iron-dependent/GI=46881237 |
| LMOf2365_1787_s_at | conserved hypothetical protein/GI=46881262 |
| LMOf2365_1793_s_at | purF amidophosphoribosyltransferase/GI=46881268 |
| LMOf2365_1836_s_at | fabD malonyl CoA-acyl carrier protein transacylase/GI=46881310 |
| LMOf2365_1889_s_at | conserved hypothetical protein/GI=46881363 |
| LMOf2365_1982_s_at | lysA diaminopimelate decarboxylase/GI=46881456 |
| LMOf2365_2018_x_at | deoC deoxyribose-phosphate aldolase/GI=46881493 |
| LMOf2365_2027_s_at | transcriptional regulator, GntR family/GI=46881502 |
| LMOf2365_2032_s_at | putative ABC transporter, permease protein/GI=46881507 |
| LMOf2365_2066_s_at | cell division protein FtsQ/GI=46881541 |
| LMOf2365_2092_s_at | conserved hypothetical protein/GI=46881567 |
| LMOf2365_2118_s_at | DNA-binding protein/GI=46881592 |
| LMOf2365_2176_s_at | transcriptional regulator, GntR family/GI=46881648 |
| LMOf2365_2342_s_at | ABC transporter, permease protein/GI=46881814 |
| LMOf2365_2517_s_at | thymidine kinase/GI=46881988 |
| LMOf2365_2565_s_at | morA morphine 6-dehydrogenase/GI=46882036 |
| LMOf2365_2658_s_at | kdpE DNA-binding response regulator KdpE/GI=46882130 |
| LMOf2365_2794_s_at | conserved hypothetical protein/GI=46882266 |
| LMOf2365_2795_s_at | conserved hypothetical protein/GI=46882267 |
| LMOf6854_0085_at | gp32, putative/GI=47016488 |
| LMOf6854_0093_s_at | conserved hypothetical protein/GI=47016496 |
| LMOf6854_0134_at | membrane protein, putative/GI=47016537 |
| LMOf6854_0146_x_at | conserved hypothetical protein/GI=47016549 |
| LMOf6854_0152_x_at | conserved hypothetical protein/GI=47014045 |
| LMOf6854_0248_s_at | gltX glutamyl-tRNA synthetase/GI=47014205 |
| LMOf6854_0263_x_at | conserved hypothetical protein/GI=47015102 |
| LMOf6854_0315_at | glycosyl hydrolase, family 1/GI=47015263 |
| LMOf6854_0323_at | conserved hypothetical protein/GI=47015193 |
| LMOf6854_0324_x_at | thiM hydroxyethylthiazole kinase/GI=47015194 |
| LMOf6854_0443_s_at | phosphate transporter family protein/GI=47015056 |
| LMOf6854_0493_s_at | hypothetical protein/GI=47016826 |
| LMOf6854_0494_x_at | hypothetical protein/GI=47016827 |
| LMOf6854_0496_s_at | conserved domain protein/GI=47016829 |
| LMOf6854_0505_s_at | conserved hypothetical protein/GI=47016838 |
| LMOf6854_0522_at | NADH:flavin oxidoreductase/GI=47016855 |
| LMOf6854_0522_x_at | NADH:flavin oxidoreductase/GI=47016855 |
| LMOf6854_0615_s_at | glycosyl hydrolase, family 1/GI=47016948 |
| LMOf6854_0744_s_at | flagellar hook protein FlgE, putative/GI=47016334 |
| LMOf6854_0770_at | methyl-accepting chemotaxis protein, putative/GI=47016360 |
| LMOf6854_0840_s_at | rarD protein/GI=47014306 |
| LMOf6854_0882_s_at | ABC transporter, ATP-binding protein/GI=47016725 |
| LMOf6854_0896_s_at | conserved hypothetical protein/GI=47016739 |
| LMOf6854_0911_at | hypothetical protein/GI=47016754 |
| LMOf6854_0946_s_at | conserved hypothetical protein/GI=47016789 |
| LMOf6854_0992_s_at | lipoprotein, putative/GI=47015377 |
| LMOf6854_1178_at | conserved hypothetical protein/GI=47016599 |
| LMOf6854_1178_x_at | conserved hypothetical protein/GI=47016599 |
| LMOf6854_1179_s_at | siroheme synthase subunit, putative/GI=47016600 |
| LMOf6854_1180_at | PduS protein/GI=47016601 |
| LMOf6854_1317_s_at | topA DNA topoisomerase I/GI=47014084 |
| LMOf6854_1624_s_at | accA acetyl-CoA carboxylase, carboxyl transferase, alpha subunit/GI=47014818 |
| LMOf6854_1656_s_at | aminopeptidase, putative/GI=47014550 |
| LMOf6854_1701_x_at | helicase, Snf2 family/GI=47014621 |
| LMOf6854_1928_at | conserved hypothetical protein/GI=47014723 |
| LMOf6854_1928_x_at | conserved hypothetical protein/GI=47014723 |
| LMOf6854_2147_at | DNA-binding protein/GI=47015799 |
| LMOf6854_2147_x_at | DNA-binding protein/GI=47015799 |
| LMOf6854_2558_s_at | phosphate ABC transporter, ATP-binding protein/GI=47015550 |
| LMOf6854_2656_at | holin, phage phi LC3 family/GI=47014863 |
| LMOf6854_2657_s_at | conserved hypothetical protein/GI=47014864 |
| LMOf6854_2749_s_at | prenyltransferase, UbiA family/GI=47013953 |
| LMOf6854_2766_s_at | PTS system, IIA component, putative/GI=47014502 |
| LMOf6854_2846_s_at | conserved hypothetical protein/GI=47016144 |
| LMOf6854_2874_s_at | hydrolase, CocENonD family/GI=47015993 |
| LMOf6854_2897_s_at | ychF GTP-binding protein YchF/GI=47016016 |
| LMOf6854_2948_s_at | beta-phosphoglucomutase/GI=47014256 |
| LMOG_00590_x_at | predicted protein |
| LMOG_00709_at | predicted protein |
| LMOG_00709_x_at | predicted protein |
| LMOG_01542_at | predicted protein |
| LMOG_01819_x_at | predicted protein |
| LMOG_01925_s_at | conserved hypothetical protein |
| LMOG_02612_at | predicted protein |
| LMOG_03106_at | DNAmethyltransferase/Pfam=PF00145.9 |
| LMOh7858_0100_s_at | pentapeptide repeats domain protein/GI=47019472 |
| LMOh7858_0417_s_at | conserved hypothetical protein/GI=47020192 |
| LMOh7858_0538_x_at | conserved hypothetical protein/GI=47019134 |
| LMOh7858_0804_at | conserved hypothetical protein/GI=47020058 |
| LMOh7858_0870_s_at | HD domain protein/GI=47017728 |
| LMOh7858_1192_x_at | conserved hypothetical protein/GI=47019636 |
| LMOh7858_2276_at | hypothetical protein/GI=47019882 |
| LMOh7858_2276_s_at | hypothetical protein/GI=47019882 |
| LMPG_02651_x_at | predicted protein |
| LMPG_03082_s_at | conserved domaincontaining protein/Pfam=PF06860.3 |
| LMSG_01585_x_at | phosphomethylpyrimidine kinase/Pfam=PF08543.4 |
| LMSG_01821_s_at | conserved hypothetical protein/Pfam=PF05043.5 |
| LMSG_02793_at | phage protein/Pfam=PF05565.3 |
| LMSG_03161_s_at | conserved hypothetical protein |
