# Supplementary material for: Genomic Characterization of Listeria monocytogenes Strains Involved in a Multistate Listeriosis Outbreak Associated with Cantaloupe in US
Source: PLoS One. 2012 Jul 31;7(7):e42448. doi: 10.1371/journal.pone.0042448 (PMC3409164; doi:10.1371/journal.pone.0042448)
Supplement: Table S2 — Probe-sets uniquely present in PC III and IV strains serotype 1/2a. (DOCX) [file pone.0042448.s002.docx]

**Supporting Information Table S2: Probe-sets uniquely present in PC III and IV strains serotype 1/2a**

| **Probe ID** | **Annotation** |
| --- | --- |
| AARI_0190_s_at | 98% similar to lmo1117 |
| AARI_0343_s_at | 99% similar to lmo0444 |
| AARI_0426_at | 98% similar to lmo0774 |
| AARI_0438_s_at | 100% similar to lmo2500 |
| AARI_0440_at | 100% similar to lmo0444 |
| AARI_0504_s_at | 99% similar to lmo2237 |
| AARI_0522_s_at | NK |
| AARI_0545_at | 99% similar to lmo1825 |
| AARI_0551_s_at | 99% similar to lmo0446 |
| AARI_0571_s_at | 100% similar to lmo2734 |
| AARI_0643_s_at | 100% similar to LMHCC_1725 |
| AARI_0695_x_at | 99% similar to lmo0107 |
| AARK_0177_s_at | 99% similar to LMHCC_2322 |
| AARK_0874_s_at | NK |
| AARK_1149_s_at | 99% similar to LMOf2365_0299 |
| AARK_1531_s_at | 99% similar to LMOf2365_1024 |
| AARK_1677_at | 99% similar to LMHCC_2212 |
| AARK_1780_x_at | 100% similar to LMOf2365_0394 |
| AARK_1899_s_at | 99% similar to LMOf2365_1790 |
| AARK_1928_s_at | 99% similar to LMOf2365_2050 |
| AARL_0174_x_at | NK |
| AARL_0331_at | NK |
| AARL_0491_s_at | NK |
| AARL_0520_s_at | NK |
| AARL_0588_s_at | NK |
| AARL_0679_s_at | NK |
| AARL_0701_s_at | 98% similar to LMHCC_1450 |
| AARL_0833_at | NK |
| AARL_0900_s_at | NK |
| AARL_0900_x_at | NK |
| AARM_0062_s_at | NK |
| AARM_0105_x_at | NK |
| AARM_0157_s_at | 99% similar to lmo2720 |
| AARM_0206_s_at | 99% similar to lmo0269 |
| AARM_0408_s_at | 100% similar to lmo0030 |
| AARM_0645_s_at | 98% similar to LMHCC_0797 |
| AARM_0699_s_at | 99% similar to lmo1512 |
| AARM_0845_s_at | 100% similar to lmo1453 |
| AARM_0869_s_at | NK |
| AARM_1167_x_at | NK |
| AARM_1201_x_at | 98% similar to LMOf2365_1121 |
| AARM_1413_s_at | 98% similar to lmo2444 |
| AARM_1440_s_at | NK |
| AARM_1460_s_at | 100% similar to lmo1258 |
| AARM_1461_s_at | 99% similar to lmo1259 |
| AARM_1501_s_at | NK |
| AARM_1646_x_at | 98% similar to lmo1861 |
| AARM_1650_x_at | NK |
| AARM_1717_x_at | NK |
| AARM_1737_x_at | 98% similar to lmo1985 |
| AARO_0340_s_at | 99% similar to LMOf2365_1597 |
| AARO_0378_at | NK |
| AARO_1179_at | NK |
| AARO_1417_s_at | 99% similar to LMOf2365_1784 |
| AARO_1609_s_at | 100% similar to LMOf2365_2475 |
| AARO_1820_x_at | 99% similar to lmo0304 |
| AARO_1856_s_at | 99% similar to lmo0448 |
| AARY_0032_x_at | 100% similar to lmo0107 |
| AARY_0039_s_at | 100% similar to lmo0121 |
| AARY_0142_x_at | 100% similar to lmo0458 |
| AARY_0249_s_at | 100% similar to lmo0915 |
| AARY_0260_at | NK |
| AARY_0261_at | NK |
| AARY_0262_at | NK |
| AARY_0316_s_at | 100% similar to lmo0563 |
| AARY_0361_s_at | 100% similar to lmo1606 |
| AARY_0396_s_at | 100% similar to lmo2143 |
| AARY_0644_x_at | 100% similar to lmo0520 |
| AARY_0668_s_at | 99% similar to lmo0535 |
| AARY_0777_s_at | 100% similar to lmo1116 |
| AARY_0821_s_at | 100% similar to lmo2839 |
| AARY_0880_s_at | 98% similar to lmo1689 |
| AARY_1012_s_at | 100% similar to lmo1513 |
| AARY_1056_s_at | 99% similar to lmo1666 |
| AARY_1084_s_at | 100% similar to lmo0444 |
| AARY_1294_s_at | 100% similar to lmo1798 |
| AARY_1307_s_at | 100% similar to lmo1479 |
| AARY_1352_x_at | 100% similar to lmo2674 |
| AARY_1544_s_at | 100% similar to lmo1513 |
| IGLMHCC_0060_at | intergenic region |
| IGLMHCC_0060_x_at | intergenic region |
| IGLMHCC_0212_x_at | intergenic region |
| IGLMHCC_0407_at | intergenic region |
| IGLMHCC_0512_at | intergenic region |
| IGLMHCC_0575_at | intergenic region |
| IGLMHCC_0575_x_at | intergenic region |
| IGLMHCC_0803_at | intergenic region |
| IGLMHCC_0997_at | intergenic region |
| IGLMHCC_0997_x_at | intergenic region |
| IGLMHCC_0999_at | intergenic region |
| IGLMHCC_1462_at | intergenic region |
| IGLMHCC_1513_s_at | intergenic region |
| IGLMHCC_1663_x_at | intergenic region |
| IGLMHCC_1703_x_at | intergenic region |
| IGLMHCC_1841_at | intergenic region |
| IGLMHCC_1996_x_at | intergenic region |
| IGLMHCC_2144_x_at | intergenic region |
| IGLMHCC_2146_x_at | intergenic region |
| IGLMHCC_2323_at | intergenic region |
| IGLMHCC_2325_at | intergenic region |
| IGLMHCC_2326_at | intergenic region |
| IGLMHCC_2350_at | intergenic region |
| IGLMHCC_2351_at | intergenic region |
| IGLMHCC_2834_at | intergenic region |
| IGlmo0105_at | intergenic region |
| IGlmo0105_x_at | intergenic region |
| IGlmo0133_at | intergenic region |
| IGlmo0133_x_at | intergenic region |
| IGlmo0135_at | intergenic region |
| IGlmo0149_s_at | intergenic region |
| IGlmo0258_at | intergenic region |
| IGlmo0304_at | intergenic region |
| IGlmo0304_x_at | intergenic region |
| IGlmo0363_at | intergenic region |
| IGlmo0363_x_at | intergenic region |
| IGlmo0364_x_at | intergenic region |
| IGlmo0365_x_at | intergenic region |
| IGlmo0378_at | intergenic region |
| IGlmo0407_x_at | intergenic region |
| IGlmo0444_at | intergenic region |
| IGlmo0445_at | intergenic region |
| IGlmo0446_at | intergenic region |
| IGlmo0447_at | intergenic region |
| IGlmo0448_at | intergenic region |
| IGlmo0449_at | intergenic region |
| IGlmo0510_at | intergenic region |
| IGlmo0559_at | intergenic region |
| IGlmo0801_at | intergenic region |
| IGlmo0908_x_at | intergenic region |
| IGlmo1031_at | intergenic region |
| IGlmo1031_x_at | intergenic region |
| IGlmo1117_x_at | intergenic region |
| IGlmo1122_x_at | intergenic region |
| IGlmo1125_at | intergenic region |
| IGlmo1125_x_at | intergenic region |
| IGlmo1140_at | intergenic region |
| IGlmo1140_x_at | intergenic region |
| IGlmo1243_at | intergenic region |
| IGlmo1243_x_at | intergenic region |
| IGlmo1253_at | intergenic region |
| IGlmo1256_at | intergenic region |
| IGlmo1256_x_at | intergenic region |
| IGlmo1257_at | intergenic region |
| IGlmo1258_at | intergenic region |
| IGlmo1258_x_at | intergenic region |
| IGlmo1261_at | intergenic region |
| IGlmo1261_x_at | intergenic region |
| IGlmo1276_s_at | intergenic region |
| IGlmo1348_at | intergenic region |
| IGlmo1600_x_at | intergenic region |
| IGlmo1613_x_at | intergenic region |
| IGlmo1796_at | intergenic region |
| IGlmo2085_x_at | intergenic region |
| IGlmo2116_x_at | intergenic region |
| IGlmo2145_at | intergenic region |
| IGlmo2145_x_at | intergenic region |
| IGlmo2365_s_at | intergenic region |
| IGlmo2392_x_at | intergenic region |
| IGlmo2394_at | intergenic region |
| IGlmo2408_at | intergenic region |
| IGlmo2411_at | intergenic region |
| IGlmo2565_x_at | intergenic region |
| IGlmo2926_at | intergenic region |
| IGLMOf2365_0314_s_at | intergenic region |
| IGLMOf2365_0383_s_at | intergenic region |
| IGLMOf2365_0485_s_at | intergenic region |
| IGLMOf2365_0500_s_at | intergenic region |
| IGLMOf2365_0501_x_at | intergenic region |
| IGLMOf2365_0502_s_at | intergenic region |
| IGLMOf2365_0517_x_at | intergenic region |
| IGLMOf2365_0638_x_at | intergenic region |
| IGLMOf2365_0810_at | intergenic region |
| IGLMOf2365_0897_x_at | intergenic region |
| IGLMOf2365_1000_at | intergenic region |
| IGLMOf2365_1121_x_at | intergenic region |
| IGLMOf2365_1129_s_at | intergenic region |
| IGLMOf2365_1751_at | intergenic region |
| IGLMOf2365_1906_x_at | intergenic region |
| IGLMOf2365_2019_x_at | intergenic region |
| IGLMOf2365_2159_at | intergenic region |
| IGLMOf2365_2234_x_at | intergenic region |
| IGLMOf2365_2236_x_at | intergenic region |
| IGLMOf2365_2289_at | intergenic region |
| IGLMOf2365_2381_s_at | intergenic region |
| IGLMOf2365_2556_x_at | intergenic region |
| IGLMOf2365_2564_s_at | intergenic region |
| IGLMOf2365_2616_x_at | intergenic region |
| IGLMOf2365_2626_x_at | intergenic region |
| IGLMOf2365_2749_x_at | intergenic region |
| IGLMOf2365_2848_s_at | intergenic region |
| Lm4b_00080a_s_at | Hypothetical protein of unknown function |
| Lm4b_00080b_s_at | Hypothetical protein of unknown function |
| Lm4b_01119_x_at | Hypothetical protein of unknown function/GI=225876179 |
| Lm4b_02561_s_at | Hypothetical protein of unknown function/GI=225877602 |
| LMBG_00131_x_at | conserved hypothetical protein |
| LMBG_02371_s_at | predicted protein |
| LMFG_01868_x_at | phage protein |
| LMFG_02839_at | conserved hypothetical protein |
| LMFG_02936_s_at | predicted protein |
| LMFG_03054_s_at | predicted protein |
| LMFG_03054_x_at | predicted protein |
| LMHCC_0718_s_at | pyrB aspartate carbamoyltransferase/GI=217333279 |
| LMHCC_0798_s_at | purN phosphoribosylglycinamide formyltransferase/GI=217333356 |
| LMHCC_0798_x_at | purN phosphoribosylglycinamide formyltransferase/GI=217333356 |
| LMHCC_0924_s_at | muramoyl-tetrapeptide carboxypeptidase family/GI=217333479 |
| LMHCC_0959_s_at | 2-Cys peroxiredoxin BAS1, (Thiol-specific antioxidant protein)/GI=217333513 |
| LMHCC_0960_s_at | aminopeptidase protein/GI=217333514 |
| LMHCC_0995_s_at | accD acetyl-CoA carboxylase, carboxyl transferase, beta subunit/GI=217333549 |
| LMHCC_0996_s_at | accA acetyl-CoA carboxylase, carboxyl transferase, alpha subunit/GI=217333550 |
| LMHCC_0997_s_at | hypothetical protein/GI=217333551 |
| LMHCC_1074_at | transcription elongation factor GreA (Transcript cleavage factor greA)(General stress protein 20M) (GSP20M)/GI=217333628 |
| LMHCC_1212_s_at | nusB transcription antitermination factor NusB/GI=217333765 |
| LMHCC_1319_s_at | treC alpha,alpha-phosphotrehalase/GI=217333870 |
| LMHCC_1319_x_at | treC alpha,alpha-phosphotrehalase/GI=217333870 |
| LMHCC_1346_at | gp30/GI=217333897 |
| LMHCC_1347_s_at | gp29/GI=217333898 |
| LMHCC_1348_s_at | gp28/GI=217333899 |
| LMHCC_1401_s_at | conserved hypothetical protein/GI=217333952 |
| LMHCC_1512_s_at | conserved hypothetical protein/GI=217334062 |
| LMHCC_1675_s_at | inner membrane transport protein YeaN/GI=217334223 |
| LMHCC_1677_s_at | late competence protein/GI=217334225 |
| LMHCC_1815_x_at | HD domain protein/GI=217334362 |
| LMHCC_2177_s_at | membrane protein, putative/GI=217334721 |
| LMHCC_2190_s_at | YtfG protein, putative/GI=217334734 |
| LMHCC_2254_x_at | DNA-binding protein IolR/GI=217334798 |
| LMHCC_2323_s_at | conserved hypothetical protein/GI=217334866 |
| LMHCC_2324_s_at | conserved hypothetical protein/GI=217334867 |
| LMHCC_2350_s_at | hypothetical protein/GI=217334893 |
| LMHCC_2351_s_at | membrane protein, putative/GI=217334894 |
| LMHCC_2489_s_at | conserved hypothetical protein/GI=217335030 |
| LMHCC_2505_s_at | EAL domain protein/GI=217335046 |
| LMHCC_2718_s_at | conserved hypothetical protein/GI=217335259 |
| LMHCC_2784_s_at | general stress protein 26 (GSP26)/GI=217335324 |
| LMHCC_2872_x_at | rpiB ribose 5-phosphate isomerase B/GI=217335409 |
| LMHCC_2899_s_at | 1,4-dihydroxy-2-naphthoate octaprenyltransferase/GI=217335436 |
| LMHG_02050_s_at | PTS system protein/Pfam=PF02378.10 |
| LMHG_02326_x_at | acetylCoA carboxylase |
| LMHG_02578_x_at | conserved hypothetical protein/Pfam=PF02618.8 |
| LMHG_02630_s_at | formate dehydrogenase/Pfam=PF04879.8 |
| LMHG_02651_x_at | phosphoribosylaminoimidazolecarboxamide formyltransferase/Pfam=PF00551.11 |
| LMHG_02737_s_at | conserved hypothetical protein |
| LMIG_00094_s_at | hydrolase/Pfam=PF00561.12 |
| LMIG_00113_s_at | conserved hypothetical protein/Pfam=PF02733.9 |
| LMIG_00356_s_at | Disomer specific 2hydroxyacid dehydrogenase/Pfam=PF02826.11 |
| LMIG_00397_s_at | antigen B |
| LMIG_00412_s_at | inositol5monophosphate dehydrogenase/Pfam=PF00571.20 |
| LMIG_00640_s_at | deoxyribosephosphate aldolase/Pfam=PF01791.1 |
| LMIG_01617_s_at | peptide chain release factor 1/Pfam=PF03462.10 |
| LMIG_01642_s_at | transcriptional regulator/Pfam=PF00532.13 |
| LMIG_01855_x_at | conserved hypothetical protein/Pfam=PF04794.4 |
| LMIG_02087_x_at | conserved hypothetical protein |
| LMIG_02163_s_at | conserved hypothetical protein/Pfam=PF09375.2 |
| LMIG_02166_s_at | conserved hypothetical protein/Pfam=PF01709.12 |
| LMIG_02311_x_at | glyoxalase/Pfam=PF00903.17 |
| LMIG_02689_x_at | conserved hypothetical protein/Pfam=PF06993.4 |
| LMIG_02874_s_at | truB |
| LMIG_02875_s_at | predicted protein |
| LMJG_00033_s_at | conserved hypothetical protein |
| LMJG_00766_x_at | ABC transporter/Pfam=PF01061.16 |
| LMKG_00550_x_at | conserved hypothetical protein/Pfam=PF07301.3 |
| LMKG_00871_at | predicted protein |
| LMKG_00871_x_at | predicted protein |
| LMKG_00998_s_at | phage protein |
| LMKG_01388_s_at | predicted protein |
| LMKG_01598_at | 6phosphobetaglucosidase |
| LMKG_01598_x_at | 6phosphobetaglucosidase |
| LMKG_01794_at | predicted protein |
| LMKG_01813_s_at | conserved hypothetical protein |
| LMKG_02058_s_at | predicted protein |
| LMKG_02149_s_at | autolysin/Pfam=PF01832.12 |
| LMKG_02734_at | predicted protein |
| LMKG_02734_s_at | predicted protein |
| LMKG_02734_x_at | predicted protein |
| LMLG_00022_s_at | predicted protein |
| LMLG_00088_at | conserved hypothetical protein |
| LMLG_00089_s_at | predicted protein |
| LMLG_00089_x_at | predicted protein |
| LMLG_00419_s_at | conserved hypothetical protein/Pfam=PF06824.3 |
| LMLG_00739_s_at | conserved hypothetical protein |
| LMLG_00751_s_at | PRDPTS system IIA 2 domain-containing protein/Pfam=PF08280.3 |
| LMLG_00788_at | conserved hypothetical protein/Pfam=PF07687.6 |
| LMLG_00805_x_at | conserved hypothetical protein/Pfam=PF08242.4 |
| LMLG_01061_s_at | precorrin3B C17methyltransferase |
| LMLG_01313_s_at | conserved hypothetical protein |
| LMLG_01353_at | predicted protein |
| LMLG_01501_s_at | alcohol dehydrogenase |
| LMLG_01504_s_at | glutamate synthase/Pfam=PF04898.6 |
| LMLG_01956_s_at | 2oxoisovalerate dehydrogenase E3/Pfam=PF07992.6 |
| LMLG_02051_s_at | malonyl CoAacyl carrier protein transacylase/Pfam=PF00698.13 |
| LMLG_02080_s_at | dihydroorotase/Pfam=PF01979.12 |
| LMLG_02235_s_at | conserved hypothetical protein |
| LMLG_02292_s_at | formate acetyltransferase |
| LMLG_02461_s_at | conserved hypothetical protein/Pfam=PF03466.12 |
| LMLG_02500_x_at | conserved hypothetical protein |
| LMLG_02550_s_at | conserved hypothetical protein/Pfam=PF01370.13 |
| LMLG_02587_s_at | conserved hypothetical protein |
| LMLG_02609_s_at | conserved hypothetical protein |
| LMLG_02754_s_at | conserved hypothetical protein/Pfam=PF00293.20 |
| LMLG_02857_x_at | conserved hypothetical protein/Pfam=PF00746.13 |
| LMMG_03049_x_at | conserved hypothetical protein |
| lmo0107_s_at | GI=16409466 |
| lmo0147_s_at | GI=16409506 |
| lmo0179_s_at | GI=16409536 |
| lmo0243_x_at | sigH RNA polymerase sigma-30 factor (sigma-H)/GI=16409608 |
| lmo0265_s_at | GI=16409630 |
| lmo0284_s_at | GI=16409649 |
| lmo0318_s_at | GI=16409682 |
| lmo0362_x_at | GI=16409740 |
| lmo0363_s_at | GI=16409741 |
| lmo0364_s_at | GI=16409742 |
| lmo0366_s_at | GI=16409744 |
| lmo0370_s_at | GI=16409748 |
| lmo0377_x_at | GI=16409755 |
| lmo0378_s_at | GI=16409756 |
| lmo0379_s_at | GI=16409757 |
| lmo0382_x_at | GI=16409760 |
| lmo0445_s_at | GI=16409822 |
| lmo0447_s_at | GI=16409824 |
| lmo0495_s_at | GI=16409871 |
| lmo0635_s_at | GI=16410024 |
| lmo0676_s_at | GI=16410065 |
| lmo0697_s_at | GI=16410086 |
| lmo0758_s_at | GI=16410147 |
| lmo0800_s_at | GI=16410189 |
| lmo0809_s_at | GI=16410198 |
| lmo0854_s_at | GI=16410257 |
| lmo0862_s_at | GI=16410265 |
| lmo0907_s_at | GI=16410310 |
| lmo0945_x_at | GI=16410347 |
| lmo0973_s_at | dltB DltB protein for D-alanine esterification of lipoteichoic acid and wall teichoic acid/GI=16410375 |
| lmo1117_x_at | GI=16410519 |
| lmo1120_s_at | GI=16410522 |
| lmo1122_s_at | GI=16410524 |
| lmo1141_s_at | GI=16410557 |
| lmo1203_s_at | cbiL GI=16410619 |
| lmo1242_s_at | GI=16410658 |
| lmo1243_s_at | GI=16410659 |
| lmo1255_s_at | GI=16410671 |
| lmo1256_s_at | GI=16410672 |
| lmo1257_s_at | GI=16410673 |
| lmo1260_s_at | proB gamma-glutamyl kinase/GI=16410676 |
| lmo1261_s_at | GI=16410677 |
| lmo1346_s_at | comGB GI=16410762 |
| lmo1347_s_at | comGA GI=16410763 |
| lmo1348_s_at | GI=16410764 |
| lmo1372_s_at | GI=16410788 |
| lmo1407_s_at | pflC pyruvate-formate lyase activating enzyme/GI=16410836 |
| lmo1427_s_at | opuCB GI=16410856 |
| lmo1453_s_at | GI=16410882 |
| lmo1479_s_at | lepA GI=16410908 |
| lmo1512_s_at | GI=16410941 |
| lmo1595_s_at | GI=16411024 |
| lmo1598_s_at | tyrS tyrosyl-tRNA synthetase/GI=16411027 |
| lmo1737_s_at | GI=16411191 |
| lmo1803_s_at | GI=16411257 |
| lmo1985_s_at | ilvN GI=16411438 |
| lmo1985_x_at | ilvN GI=16411438 |
| lmo2018_s_at | GI=16411471 |
| lmo2036_s_at | murD GI=16411506 |
| lmo2061_s_at | GI=16411531 |
| lmo2143_s_at | GI=16411613 |
| lmo2144_s_at | GI=16411614 |
| lmo2345_s_at | GI=16411833 |
| lmo2362_s_at | GI=16411850 |
| lmo2364_s_at | Hypothetical protein/GI=16411852 |
| lmo2371_s_at | GI=16411859 |
| lmo2374_s_at | GI=16411862 |
| lmo2405_x_at | GI=16411893 |
| lmo2408_at | GI=16411896 |
| lmo2409_at | GI=16411897 |
| lmo2410_at | GI=16411898 |
| lmo2497_s_at | GI=16411985 |
| lmo2503_s_at | GI=16411991 |
| lmo2583_s_at | GI=16412071 |
| lmo2592_s_at | GI=16412080 |
| lmo2755_s_at | GI=16412255 |
| LMOf2365_0253_s_at | RNA methyltransferase, TrmH family, group 3/GI=46879738 |
| LMOf2365_0313_s_at | hypothetical protein/GI=46879799 |
| LMOf2365_0480_s_at | putative transcriptional regulator/GI=46879962 |
| LMOf2365_0500_s_at | hypothetical protein/GI=46879982 |
| LMOf2365_0501_s_at | hypothetical protein/GI=46879983 |
| LMOf2365_0504_x_at | HD domain protein/GI=46879986 |
| LMOf2365_0519_s_at | NADH:flavin oxidoreductase/GI=46880000 |
| LMOf2365_0651_x_at | conserved hypothetical protein/GI=46880133 |
| LMOf2365_0670_s_at | putative transcriptional regulator/GI=46880151 |
| LMOf2365_0828_x_at | HD domain protein/GI=46880308 |
| LMOf2365_0883_s_at | hypothetical protein/GI=46880364 |
| LMOf2365_0904_s_at | acpS holo-(acyl-carrier-protein) synthase/GI=46880385 |
| LMOf2365_0919_s_at | conserved hypothetical protein/GI=46880399 |
| LMOf2365_0920_s_at | hypothetical protein/GI=46880400 |
| LMOf2365_1065_s_at | moaE molybdenum cofactor biosynthesis protein E/GI=46880543 |
| LMOf2365_1126_s_at | conserved hypothetical protein/GI=46880603 |
| LMOf2365_1127_s_at | conserved hypothetical protein/GI=46880604 |
| LMOf2365_1170_s_at | pduM propanediol utilization protein PduM/GI=46880647 |
| LMOf2365_1172_s_at | PduO protein/GI=46880649 |
| LMOf2365_1228_s_at | conserved hypothetical protein/GI=46880706 |
| LMOf2365_1298_s_at | transcriptional regulator CodY/GI=46880775 |
| LMOf2365_1466_s_at | zurA2 zinc ABC transporter, ATP-binding protein/GI=46880943 |
| LMOf2365_1759_s_at | gltC transcriptional regulator GltC/GI=46881235 |
| LMOf2365_1789_s_at | purD phosphoribosylamine--glycine ligase/GI=46881264 |
| LMOf2365_2227_s_at | oligopeptide ABC transporter, permease protein/GI=46881699 |
| LMOf2365_2261_s_at | putative membrane protein/GI=46881733 |
| LMOf2365_2381_s_at | conserved hypothetical protein/GI=46881853 |
| LMOf2365_2469_s_at | phosphate ABC transporter, ATP-binding protein/GI=46881940 |
| LMOf2365_2567_at | hypothetical protein/GI=46882039 |
| LMOf2365_2567_x_at | hypothetical protein/GI=46882039 |
| LMOf2365_2607_s_at | cobalt transport protein/GI=46882079 |
| LMOf2365_2714_s_at | conserved hypothetical protein/GI=46882186 |
| LMOf2365_2724_s_at | transcriptional regulator, LacI family/GI=46882196 |
| LMOf6854_0134_s_at | membrane protein, putative/GI=47016537 |
| LMOf6854_0152_s_at | conserved hypothetical protein/GI=47014045 |
| LMOf6854_0154_s_at | conserved hypothetical protein/GI=47014091 |
| LMOf6854_0157_x_at | conserved hypothetical protein/GI=47014094 |
| LMOf6854_0158_at | conserved hypothetical protein/GI=47014095 |
| LMOf6854_0193_x_at | oligo-1,6-glucosidase/GI=47015425 |
| LMOf6854_0401_s_at | Iron permease FTR1 family family/GI=47014950 |
| LMOf6854_0416_at | hypothetical protein/GI=47015029 |
| LMOf6854_0416_x_at | hypothetical protein/GI=47015029 |
| LMOf6854_0417_x_at | conserved hypothetical protein/GI=47015030 |
| LMOf6854_0502_x_at | conserved hypothetical protein/GI=47016835 |
| LMOf6854_0597_x_at | phosphoglycerate mutase family protein/GI=47016930 |
| LMOf6854_0618_s_at | membrane protein, putative/GI=47014735 |
| LMOf6854_0856_s_at | HD domain protein/GI=47016699 |
| LMOf6854_0909_x_at | conserved hypothetical protein/GI=47016752 |
| LMOf6854_0977_x_at | conserved hypothetical protein/GI=47015362 |
| LMOf6854_1105_s_at | pdhB pyruvate dehydrogenase complex, E1 component, pyruvate dehydrogenase beta subunit/GI=47015491 |
| LMOf6854_1155_s_at | conserved hypothetical protein/GI=47016577 |
| LMOf6854_1294_x_at | hypothetical protein/GI=47014581 |
| LMOf6854_1295_at | conserved hypothetical protein/GI=47014582 |
| LMOf6854_1295_x_at | conserved hypothetical protein/GI=47014582 |
| LMOf6854_1582_s_at | L-lactate dehydrogenase/GI=47013917 |
| LMOf6854_1592_s_at | ribonuclease G/GI=47014890 |
| LMOf6854_1628_s_at | CBS domain protein/GI=47014822 |
| LMOf6854_1789_s_at | ABC transporter, permease protein/GI=47015942 |
| LMOf6854_1795_s_at | alcohol dehydrogenase, iron-dependent/GI=47015948 |
| LMOf6854_1854_s_at | glutamine amidotransferase, class-I/GI=47014031 |
| LMOf6854_1941_at | hypothetical protein/GI=47013989 |
| LMOf6854_1972_s_at | conserved hypothetical protein/GI=47015149 |
| LMOf6854_1990_s_at | hepB heptaprenyl diphosphate synthase, component II/GI=47013897 |
| LMOf6854_2065_s_at | transcriptional regulator, GntR family/GI=47014968 |
| LMOf6854_2111_s_at | conserved hypothetical protein/GI=47014353 |
| LMOf6854_2405_s_at | amino acid ABC transporter, ATP-binding protein/GI=47015641 |
| LMOf6854_2688_s_at | gp55/GI=47014132 |
| LMOf6854_2712_s_at | cas2 CRISPR-associated protein Cas2/GI=47014481 |
| LMOf6854_2713_s_at | cas1 CRISPR-associated protein Cas1/GI=47014482 |
| LMOf6854_2715_s_at | conserved hypothetical protein/GI=47014484 |
| LMOf6854_2759_s_at | conserved hypothetical protein/GI=47014495 |
| LMOf6854_2817_s_at | phosphosugar-binding transcriptional regulator, RpiR family/GI=47016115 |
| LMOf6854_2849_s_at | HAD-superfamily hydrolase, subfamily IA, variant 1/GI=47016147 |
| LMOf6854_2953_s_at | alcohol dehydrogenase, zinc-dependent/GI=47014261 |
| LMOG_00938_s_at | hypothetical protein similar to internalin |
| LMOG_01931_s_at | hypothetical protein similar to internalin/Pfam=PF08191.3 |
| LMOG_03220_x_at | conserved hypothetical protein/Pfam=PF00746.13 |
| LMOh7858_0339_s_at | conserved hypothetical protein/GI=47020119 |
| LMOh7858_0850_s_at | rarD protein/GI=47017830 |
| LMOh7858_1186_s_at | conserved hypothetical protein/GI=47019631 |
| LMOh7858_1187_s_at | conserved hypothetical protein/GI=47019632 |
| LMOh7858_1833_s_at | map methionine aminopeptidase, type I/GI=47017382 |
| LMOh7858_2088_s_at | iron compound ABC transporter, permease protein/GI=47017007 |
| LMOh7858_2508_at | hypothetical protein/GI=47019248 |
| LMPG_03052_s_at | conserved hypothetical protein/Pfam=PF00746.13 |
| LMRG_00002_at | threonine aldolase family protein/Pfam=PF01212.13 |
| LMRG_00003_at | conserved hypothetical protein/Pfam=PF07252.3 |
| LMRG_00004_at | conserved hypothetical protein |
| LMRG_00006_at | conserved hypothetical protein/Pfam=PF07510.3 |
| LMRG_00007_x_at | conserved hypothetical protein/Pfam=PF04794.4 |
| LMRG_00011_at | thiaminephosphate pyrophosphorylase/Pfam=PF02581.9 |
| LMRG_00021_at | conserved hypothetical protein/Pfam=PF06458.4 |
| LMRG_00022_x_at | conserved hypothetical protein |
| LMRG_00029_at | conserved hypothetical protein/Pfam=PF06860.3 |
| LMRG_00033_x_at | transketolase/Pfam=PF02780.12 |
| LMRG_00058_at | ywbM/Pfam=PF09375.2 |
| LMRG_00058_x_at | ywbM/Pfam=PF09375.2 |
| LMRG_00059_s_at | ipa29d/Pfam=PF04261.4 |
| LMRG_00075_x_at | IolR/Pfam=PF08279.4 |
| LMRG_00094_at | glycosyl hydrolase/Pfam=PF07748.5 |
| LMRG_00151_at | predicted protein |
| LMRG_00152_at | predicted protein |
| LMRG_00170_x_at | NADH:flavin oxidoreductase/Pfam=PF00724.12 |
| LMRG_00270_at | deoxyribodipyrimidine photolyase/Pfam=PF03441.6 |
| LMRG_00325_at | conserved hypothetical protein |
| LMRG_00325_x_at | conserved hypothetical protein |
| LMRG_00366_at | fliR/Pfam=PF01311.12 |
| LMRG_00386_at | flagellar hook protein FlgE/Pfam=PF07559.6 |
| LMRG_00397_at | conserved hypothetical protein/Pfam=PF02561.6 |
| LMRG_00397_x_at | conserved hypothetical protein/Pfam=PF02561.6 |
| LMRG_00457_s_at | conserved hypothetical protein |
| LMRG_00499_at | conserved hypothetical protein |
| LMRG_00560_x_at | predicted protein/Pfam=PF00903.17 |
| LMRG_00561_x_at | conserved hypothetical protein |
| LMRG_00567_s_at | conserved hypothetical protein |
| LMRG_00605_x_at | conserved hypothetical protein |
| LMRG_00629_at | conserved hypothetical protein |
| LMRG_00645_at | precorrin3B C17methyltransferase/Pfam=PF00590.12 |
| LMRG_00650_x_at | cbiM/Pfam=PF01891.8 |
| LMRG_00651_at | cobalt transport protein CbiN/Pfam=PF02553.7 |
| LMRG_00663_at | conserved hypothetical protein/Pfam=PF05343.6 |
| LMRG_00930_at | oxidoreductase family protein/Pfam=PF01370.13 |
| LMRG_00931_at | transcription regulator/Pfam=PF00376.15 |
| LMRG_01008_at | conserved hypothetical protein |
| LMRG_01008_x_at | conserved hypothetical protein |
| LMRG_01022_at | ABC transporter/Pfam=PF00005.19 |
| LMRG_01061_at | conserved hypothetical protein/Pfam=PF00563.12 |
| LMRG_01069_at | conserved hypothetical protein/Pfam=PF07719.9 |
| LMRG_01070_at | 3phosphoshikimate 1carboxyvinyltransferase/Pfam=PF00275.12 |
| LMRG_01132_at | ilvB/Pfam=PF02776.10 |
| LMRG_01133_at | ilvN/Pfam=PF01842.17 |
| LMRG_01172_at | Laspartate oxidase/Pfam=PF07992.6 |
| LMRG_01182_x_at | ftsA/Pfam=PF02491.12 |
| LMRG_01275_at | glycosyl transferase/Pfam=PF03636.7 |
| LMRG_01294_x_at | menB/Pfam=PF00378.12 |
| LMRG_01301_at | predicted protein/Pfam=PF02494.8 |
| LMRG_01308_s_at | predicted protein |
| LMRG_01710_s_at | UDPNacetylglucosamine 2epimerase/Pfam=PF02350.11 |
| LMRG_01712_x_at | ATP synthase protein I/Pfam=PF05468.3 |
| LMRG_01751_x_at | phosphate ABC transporter/Pfam=PF00528.14 |
| LMRG_01790_at | phosphoglycerate kinase/Pfam=PF00162.11 |
| LMRG_01796_at | est2 |
| LMRG_01801_s_at | conserved hypothetical protein/Pfam=PF01381.14 |
| LMRG_01804_at | 6glucosyltransferase/Pfam=PF03422.7 |
| LMRG_01837_at | FeS assembly protein SufB/Pfam=PF01458.9 |
| LMRG_01841_at | conserved hypothetical protein |
| LMRG_01872_at | major facilitator family transporter/Pfam=PF07690.8 |
| LMRG_01873_at | phosphoserine aminotransferase/Pfam=PF00266.11 |
| LMRG_01876_at | conserved hypothetical protein |
| LMRG_01876_x_at | conserved hypothetical protein |
| LMRG_01890_x_at | conserved hypothetical protein/Pfam=PF07006.3 |
| LMRG_01891_at | conserved hypothetical protein/Pfam=PF07006.3 |
| LMRG_01892_at | conserved hypothetical protein |
| LMRG_01962_at | alphamannosidase/Pfam=PF01074.14 |
| LMRG_02005_at | conserved hypothetical protein |
| LMRG_02020_s_at | conserved hypothetical protein/Pfam=PF03773.5 |
| LMRG_02080_s_at | ABC transporter/Pfam=PF01061.16 |
| LMRG_02114_at | glycine betaineL-proline ABC transporter/Pfam=PF00571.20 |
| LMRG_02118_s_at | CutC family protein/Pfam=PF03932.6 |
| LMRG_02134_x_at | conserved hypothetical protein |
| LMRG_02138_at | conserved hypothetical protein/Pfam=PF01844.15 |
| LMRG_02193_x_at | phosphotriesterase family protein/Pfam=PF02126.10 |
| LMRG_02206_s_at | ribulosephosphate 3epimerase family protein/Pfam=PF00834.11 |
| LMRG_02207_x_at | ribose 5phosphate isomerase B/Pfam=PF02502.10 |
| LMRG_02219_at | rpiB/Pfam=PF02502.10 |
| LMRG_02220_at | conserved hypothetical protein |
| LMRG_02220_x_at | conserved hypothetical protein |
| LMRG_02221_at | ImpBMucBSamB family protein/Pfam=PF00817.12 |
| LMRG_02221_s_at | ImpBMucBSamB family protein/Pfam=PF00817.12 |
| LMRG_02278_at | D-alanine-D-alanine ligase/Pfam=PF07478.5 |
| LMRG_02287_x_at | conserved hypothetical protein/Pfam=PF04041.5 |
| LMRG_02380_at | EAL domaincontaining protein/Pfam=PF00563.12 |
| LMRG_02380_x_at | EAL domaincontaining protein/Pfam=PF00563.12 |
| LMRG_02404_at | surface anchored protein/Pfam=PF05738.5 |
| LMRG_02419_at | Lrhamnose isomerase/Pfam=PF06134.3 |
| LMRG_02481_x_at | conserved hypothetical protein/Pfam=PF02272.11 |
| LMRG_02503_s_at | amidophosphoribosyltransferase/Pfam=PF00310.13 |
| LMRG_02572_at | conserved hypothetical protein |
| LMRG_02573_at | transcriptional regulator/Pfam=PF03466.12 |
| LMRG_02574_at | acetyltransferase/Pfam=PF00583.16 |
| LMRG_02575_at | conserved hypothetical protein/Pfam=PF04851.7 |
| LMRG_02576_at | N6 DNA methylase/Pfam=PF02384.8 |
| LMRG_02577_at | type I restriction enzyme S protein/Pfam=PF01420.11 |
| LMRG_02578_at | hypothetical protein/Pfam=PF02590.8 |
| LMRG_02578_x_at | hypothetical protein/Pfam=PF02590.8 |
| LMRG_02593_at | conserved hypothetical protein |
| LMRG_02594_at | conserved hypothetical protein/Pfam=PF00005.19 |
| LMRG_02597_at | ABC transporter/Pfam=PF08402.2 |
| LMRG_02708_at | arginyltRNA synthetase/Pfam=PF05746.7 |
| LMRG_02710_at | CTP synthase/Pfam=PF06418.6 |
| LMRG_02759_s_at | glutamate1semialdehyde 2,1aminomutase/Pfam=PF00202.13 |
| LMRG_02784_x_at | scrK/Pfam=PF00480.12 |
| LMRG_02794_s_at | conserved hypothetical protein/Pfam=PF00999.13 |
| LMRG_02837_at | dihydrouridine synthase family protein/Pfam=PF01207.9 |
| LMRG_02837_x_at | dihydrouridine synthase family protein/Pfam=PF01207.9 |
| LMRG_02862_at | conserved hypothetical protein |
| LMRG_02863_at | conserved hypothetical protein/Pfam=PF06860.3 |
| LMRG_02864_at | conserved hypothetical protein |
| LMRG_02864_s_at | conserved hypothetical protein |
| LMRG_02872_x_at | conserved hypothetical protein |
| LMRG_02887_at | predicted protein |
| LMRG_02891_at | conserved hypothetical protein/Pfam=PF08937.3 |
| LMRG_02892_at | predicted protein |
| LMRG_02893_at | predicted protein |
| LMRG_02893_x_at | predicted protein |
| LMRG_02907_at | conserved hypothetical protein |
| LMRG_02908_at | conserved hypothetical protein |
| LMRG_02910_at | conserved hypothetical protein |
| LMRG_02933_at | conserved hypothetical protein |
| LMRG_02934_at | resolvase |
| LMRG_02935_at | cassette chromosome recombinase B/Pfam=PF07508.5 |
| LMRG_02937_at | predicted protein |
| LMRG_02937_s_at | predicted protein |
| LMSG_00232_x_at | tRNA uridine 5carboxymethylaminomethyl modification enzyme GidA/Pfam=PF01134.14 |
| LMSG_02867_x_at | naphthoate synthase/Pfam=PF00378.12 |
