# Supplementary material for: Genomic Characterization of Listeria monocytogenes Strains Involved in a Multistate Listeriosis Outbreak Associated with Cantaloupe in US
Source: PLoS One. 2012 Jul 31;7(7):e42448. doi: 10.1371/journal.pone.0042448 (PMC3409164; doi:10.1371/journal.pone.0042448)
Supplement: Table S3 — Probe-sets uniquely present in PC IV serotype 1/2a.1. (DOCX) [file pone.0042448.s003.docx]

**Supporting Information Table S3: Probe-sets uniquely present in PC IV serotype 1/2a**

| Probe ID | Annotation |
| --- | --- |
| AARI_0014_at | NK |
| AARI_0606_at | NK |
| AARO_0405_x_at | NK |
| IGLMHCC_2942_s_at | intergenic region |
| IGLMHCC_2950_s_at | intergenic region |
| IGLMHCC_2953_at | intergenic region |
| IGLMHCC_2983_s_at | intergenic region |
| IGlmo2271_at | intergenic region |
| IGlmo2290_at | intergenic region |
| IGlmo2290_x_at | intergenic region |
| IGlmo2302_x_at | intergenic region |
| IGlmo2303_at | intergenic region |
| IGlmo2328_x_at | intergenic region |
| IGlmo2330_at | intergenic region |
| IGlmo2330_x_at | intergenic region |
| LMBG_01625_s_at | conserved hypothetical protein |
| LMBG_01658_s_at | TerS/Pfam=PF03592.8 |
| LMBG_01659_s_at | TerL/Pfam=PF07570.4 |
| LMBG_01666_s_at | phage protein |
| LMBG_01681_s_at | peptidoglycan lytic enzyme |
| LMBG_01688_s_at | conserved hypothetical protein |
| LMFG_02401_s_at | conserved hypothetical protein/Pfam=PF01381.14 |
| LMFG_02402_at | predicted protein/Pfam=PF01381.14 |
| LMFG_02517_at | predicted protein |
| LMFG_02518_s_at | predicted protein |
| LMFG_02983_s_at | phage protein |
| LMFG_02989_s_at | phage protein/Pfam=PF06152.3 |
| LMFG_03002_s_at | phage protein/Pfam=PF06997.3 |
| LMFG_03003_s_at | phage protein/Pfam=PF06605.3 |
| LMFG_03181_s_at | phage protein |
| LMFG_03194_s_at | conserved hypothetical protein/Pfam=PF03837.6 |
| LMFG_03210_at | predicted protein |
| LMHCC_2946_s_at | conserved hypothetical protein/GI=217335483 |
| LMHCC_2953_s_at | gp47/GI=217335490 |
| LMHCC_2977_s_at | protein gp66/GI=217335514 |
| LMHCC_2983_s_at | putative scaffolding protein/GI=217335520 |
| LMHCC_2983_x_at | putative scaffolding protein/GI=217335520 |
| LMHCC_2984_s_at | phage coat protein/GI=217335521 |
| LMHCC_2987_s_at | Gp10/GI=217335524 |
| LMJG_02933_x_at | conserved hypothetical protein |
| LMJG_02938_s_at | phage protein |
| lmo2279_s_at | holin [Bacteriophage A118]/GI=16411749 |
| lmo2281_s_at | protein gp22 [Bacteriophage A118]/GI=16411751 |
| lmo2282_s_at | protein gp21 [Bacteriophage A118]/GI=16411752 |
| lmo2288_s_at | Protein gp15 [Bacteriophage A118]/GI=16411758 |
| lmo2304_s_at | Bacteriophage A118 gp65 protein/GI=16411774 |
| lmo2305_s_at | GI=16411775 |
| lmo2322_s_at | gp44 [Bacteriophage A118]/GI=16411811 |
| lmo2325_at | GI=16411814 |
| lmo2326_s_at | GI=16411815 |
| lmo2332_s_at | int putative integrase [Bacteriophage A118]/GI=16411821 |
| LMOf6854_2342_s_at | protein gp23/GI=47015579 |
| LMOf6854_2357_s_at | protein gp8/GI=47015594 |
| LMOf6854_2362_s_at | phage portal protein, putative, A118 family/GI=47015599 |
| LMOf6854_2365_s_at | gp68/GI=47015602 |
| LMOf6854_2380_s_at | DnaD domain protein/GI=47015617 |
| LMOf6854_2654_at | gp27/GI=47014861 |
| LMOf6854_2697_x_at | conserved hypothetical protein/GI=47014140 |
| LMRG_01511_at | U153 integrase/Pfam=PF07508.5 |
| LMRG_01511_x_at | U153 integrase/Pfam=PF07508.5 |
| LMRG_01512_at | conserved hypothetical protein/Pfam=PF04326.6 |
| LMRG_01513_at | predicted protein |
| LMRG_01514_at | phage protein/Pfam=PF01381.14 |
| LMRG_01521_at | phage protein |
| LMRG_01521_s_at | phage protein |
| LMRG_01521_x_at | phage protein |
| LMRG_01523_x_at | phage protein |
| LMRG_01527_x_at | Gp32 protein |
| LMRG_01531_at | TerS/Pfam=PF03592.8 |
| LMRG_01532_at | TerL/Pfam=PF07570.4 |
| LMRG_01532_x_at | TerL/Pfam=PF07570.4 |
| LMRG_01533_at | phage protein/Pfam=PF05126.4 |
| LMRG_01534_at | phage protein/Pfam=PF06152.3 |
| LMRG_01534_x_at | phage protein/Pfam=PF06152.3 |
| LMRG_01537_x_at | phage protein |
| LMRG_01538_at | phage protein |
| LMRG_01538_s_at | phage protein |
| LMRG_01538_x_at | phage protein |
| LMRG_01539_at | phage protein |
| LMRG_01539_x_at | phage protein |
| LMRG_01540_at | phage protein |
| LMRG_01540_x_at | phage protein |
| LMRG_01541_at | major tail shaft protein |
| LMRG_01541_x_at | major tail shaft protein |
| LMRG_01542_s_at | phage protein/Pfam=PF02368.10 |
| LMRG_01542_x_at | phage protein/Pfam=PF02368.10 |
| LMRG_01543_at | phage protein |
| LMRG_01544_at | phage protein/Pfam=PF06854.3 |
| LMRG_01545_at | tmp |
| LMRG_01546_at | phage protein/Pfam=PF06997.3 |
| LMRG_01548_s_at | phage protein |
| LMRG_01550_at | phage protein |
| LMRG_01550_x_at | phage protein |
| LMRG_01554_at | LalanylDglutamate peptidase |
| LMRG_01554_x_at | LalanylDglutamate peptidase |
| LMRG_01557_at | predicted protein |
| LMRG_01558_at | Gp22 protein |
| LMRG_01558_x_at | Gp22 protein |
| LMRG_02918_x_at | predicted protein |
| LMRG_02920_s_at | phage protein/Pfam=PF08346.4 |
| LMRG_02920_x_at | phage protein/Pfam=PF08346.4 |
| LMRG_02921_at | sugarphospahte nucleotidyltransferase |
| LMSG_02941_s_at | TerL |
| LMSG_03002_s_at | recombinase/Pfam=PF03837.6 |
| LMSG_03154_x_at | phage protein |
| LMSG_03167_s_at | predicted protein |
| LMSG_03174_s_at | phage protein/Pfam=PF03374.6 |
| LMSG_03174_x_at | phage protein/Pfam=PF03374.6 |
